# eHealth Interventions to Support Self-Management in People With Musculoskeletal Disorders, “eHealth: It’s TIME”—A Scoping Review

**DOI:** 10.1093/ptj/pzab307

**Published:** 2022-01-13

**Authors:** Marie Kelly, Brona Fullen, Denis Martin, Sinéad McMahon, Joseph G McVeigh

**Affiliations:** 1 Discipline of Physiotherapy, School of Clinical Therapies, College of Medicine and Health, University College Cork, Cork, Ireland; 2 Department of Physiotherapy, Mercy University Hospital, Cork, Ireland; 3 School of Public Health, Physiotherapy and Sports Science, University College Dublin, Dublin, Ireland; 4 School of Health and Life Sciences, Teesside University, Middlesbrough, United Kingdom; 5 NIHR Applied Research Collaborative, North East and North Cumbria, United Kingdom

**Keywords:** Digital Health, eHealth, Musculoskeletal Pain, Review, Self-Management

## Abstract

**Objective:**

eHealth-mediated interventions have been proposed as one option to support self-management in those with musculoskeletal disorders (MSDs). This scoping review aimed to chart the evidence regarding eHealth modalities, musculoskeletal diagnosis, and outcomes of eHealth-mediated self-management support interventions in persons with MSDs and identify any gaps within the literature.

**Methods:**

Six electronic databases (MEDLINE, CINAHL, PsycINFO, Embase, Scopus, and the Cochrane Database of Systematic Reviews), 7 grey literature sources (eg, OpenGrey), and reference and citation lists of included studies were searched from database inception to July 2020. Published studies of adult participants with a MSD utilizing an eHealth intervention to support self-management were included. Studies were limited to those published in English. Two reviewers independently screened all studies. Data were extracted by 1 reviewer and reviewed by another reviewer.

**Results:**

After screening 3377 titles and abstracts followed by 176 full texts, 87 studies fulfilled the eligibility criteria. The majority were published in the last 5 years (n = 48; 55%), with almost one-third originating in the United States (n = 28; 32%). The most common eHealth modality type was internet based (n = 22; 35%), with almost one-half (n = 41; 47%) of the included studies involving participants with widespread musculoskeletal symptoms. The most commonly reported outcomes were related to body functions (ie, pain intensity) (n = 67; 45%), closely followed by activities and participation (ie, function) (n = 65; 44%), with environmental factors (ie, health care utilization) the least commonly reported (n = 17; 20%).

**Conclusions:**

There is considerable variation within the eHealth-mediated self-management support intervention literature. Research is needed on the role of eHealth-mediated self-management support interventions across a broad range of MSDs to guide clinical practice.

**Impact:**

This scoping review has identified gaps in the literature relating to specific eHealth modalities, musculoskeletal diagnoses, and health care utilization data, which should guide future research.

## Introduction

The profound burden of disease associated with musculoskeletal disorders (MSDs) is well established. Accounting for one-fifth of the world’s total “years lived with disability,”^1^ MSDs such as knee osteoarthritis and back pain also represent a significant economic cost.^2^ This global burden is expected to rise in the coming decades due to an increase in related factors such as obesity, population growth, and aging.^3^ Health care systems worldwide face the challenge of how to manage the rising demand with limited resources.

Against this background, eHealth has attracted considerable interest as a possible solution. Definitions of eHealth are many and varied^4^; in this paper, an eHealth modality is defined as any specific technology applied in the context of health care.^4^ Examples include internet-based interventions, telephone support (interventions with telephone support from health practitioners), interactive voice response (the use of a phone’s touch-tone keypad to provide responses to automated scripts), virtual reality (a 3-dimensional computer-generated environment the individual can explore, interact with, and manipulate), and mobile phone applications (mobile-based or mobile-enhanced programs) that deliver health-related services. Telehealth is an example of eHealth,^5^ and with respect to telehealth, this scoping review focuses on “real-time” telehealth (videoconferencing and telephone-based interventions).^6^ More detailed definitions are available in Supplementary File.

eHealth modalities have the potential to improve the availability of health care services, helping individuals to access services and support from their own home. Furthermore, eHealth modalities may provide more cost-effective treatment options, reducing the requirement for travel and direct health care professional involvement, although actual savings are unknown at present.^7^ Such modalities also have the potential to improve treatment durability, because individuals can obtain support and reinforcement of skills after formal treatment has ceased.^7^ The COVID-19 pandemic, viewed as a “black swan” moment,^8–10^ appears to have been a significant catalyst for the temporary implementation of eHealth modalities into routine practice. However, much of the literature on eHealth predates the COVID-19 pandemic and is very fragmented, with existing systematic reviews mainly involving those with a chronic MSD^11^^,^^12^ and often limited to a specific eHealth subgroup such as internet-based^12^ or mobile phone applications.^11^

Self-management strategies for people with MSDs are complex interventions that involve patient education and behavior modification and are designed to encourage people to take an active self-management role to improve health outcomes.^13^ Effective self-management interventions have been identified as a priority implementation area for health care services.^14^ Supported self-management aims to empower people to take an active role in managing their condition and health behaviors collaboratively with their health care professional.^15^ Supported self-management is an important step toward better health care provision, which results in positive outcomes including reduced pain and improved self-efficacy.^16^ eHealth may be used to promote and enable self-management support on a large scale; however, uptake may be limited to those with knowledge and access to appropriate technology and hence may exacerbate health care inequality.^17^ Another concern is that eHealth modalities may increase dependence on health care professionals.^18^

Self-management and its support comprise a wide range of potential activities and interventions, with no single component identified as being more important than another.^19^ Taylor et al^19^ suggest that the detail and quality of reporting of complex self-management interventions was a barrier to understanding their effective components and wider implementation,^19^ which contributed to the development of the Practical Review in Self-Management Support (PRISMS) taxonomy.^20^ This taxonomy identified 14 separate elements, which are considered important for self-management support by individuals and caregivers^21^ (eg, information provision and patient education; remote monitoring with feedback and action plans).^22^ eHealth has the potential to contribute to many of these components.

The PRISMS taxonomy has previously been used in evidence syntheses for other conditions^22^^,^^23^; however, to our knowledge, there has not been a review in the MSD literature that outlines components of self-management support with reference to the PRISMS taxonomy.

Given the variety of eHealth options and the varied nature of self-management interventions, this review aimed to provide a broad overview of the evidence for eHealth-mediated self-management support interventions using scoping review methodology. This methodology is recommended when mapping literature predicted to be large and heterogeneous.^24^ To characterize the available literature and identify any gaps or limitations, this scoping review had the following objectives: (1) to map the available evidence base on eHealth modalities, musculoskeletal diagnosis, and outcomes; and (2) to chart intervention characteristics, such as intervention provider, duration, and frequency, together with information on outcomes described as improved, unchanged, or worse.

## Methods

### Scoping Review

This scoping review followed the Joanna Briggs Institute methodology for scoping reviews^24^ and reported findings utilizing the elements provided in the Preferred Reporting Items for Systematic reviews and meta-analyses extension for scoping reviews.^25^ Our protocol was registered with the Open Science Framework (https://osf.io/29rd6) and published.^26^ Because our full methods are available in our protocol, they are outlined briefly below.

### Data Sources and Searches

For MEDLINE (via EBSCO), a 3-step search strategy was utilized as outlined in the protocol.^26^ One reviewer (M.K.) developed the search strategy, with assistance from 2 professional librarians. A comprehensive electronic search of MEDLINE (EBSCO), CINAHL (EBSCO), PsycINFO (EBSCO), Embase, Scopus, and the Cochrane Database of Systematic Reviews databases was conducted from inception to July 2020. Studies were limited to those published in English. The main literature search was supplemented by utilizing the Canadian Agency for Drugs and Technology Gray Matters approach^27^ with Google Scholar, Health Technology Assessment agencies (Canada, Australia, Ireland, UK, and USA) along with OpenGrey searched for grey literature. Additionally, screening of citation and reference lists of included studies was conducted to identify other potential studies that met the inclusion criteria.

### Study Selection

Literature search results were screened using Covidence online software (http://www.covidence.org). Titles and abstracts were screened by 2 review authors (M.K. and S.M.) independently against the eligibility criteria (Suppl. Table 1). Any disagreements were resolved by consensus or by decision of a third reviewer (J.M.). Studies that met the inclusion criteria were then retrieved in full and independently assessed in detail against the eligibility criteria by 2 review authors (M.K. and S.M.). Any conflicts were resolved by consensus or a third reviewer (J.M.).

**Table 1:** Characteristics of the Studies Included in Scoping Review^a^

**Characteristics**	**Number of Studies With Characteristic (N = 87)**
Study design	
Randomized controlled trial	60
Randomized non-inferiority trial	2
Observational	10
Qualitative	8
Mixed methods	7
Origin of study	
United States	28
Australia	16
Sweden	11
United Kingdom	6
Norway	5
Germany	5
Netherlands	4
China	3
Nigeria	2
Switzerland	2
Austria	1
Canada	1
India	1
Italy	1
South Korea	1
Year of publication	
2016–2020	49
2011–2015	26
2006–2010	5
Prior to 2006	7
Musculoskeletal diagnosis	
Widespread musculoskeletal conditions^***a***^	41
Back pain	27
Osteoarthritis	12
Knee pain	5
Neck pain	2
Duration of symptoms	
Chronic (>12 wk)	79
Acute (<12 wk)	4
Unspecified duration	3

*^a^* Indicates pain at multiple sites.

### Data Extraction

Data extraction was conducted using a standardized data extraction form that was developed a priori based on the Joanna Briggs Institute data extraction tool^24^ and the template for Intervention Description and Replication checklist.^28^ Data charted are synthesized in Tables 1 to 4 and in Supplementary Table 2. Data were charted for all studies by the lead author (M.K.) and checked by another author for accuracy, with any discrepancies resolved by consultation.

### Data Synthesis and Analysis

Quantitative and qualitative data are presented in tabular format and synthesized narratively, prioritizing information reporting on distribution of studies by eHealth modality, musculoskeletal diagnosis, and quantitative and qualitative outcomes. We mapped the interventions in included studies to the components of the PRISMS taxonomy of self-management support.^20^ Quantitative outcomes are presented in line with the International Classification of Functioning, Disability and Health framework,^29^ with overall study results categorized descriptively (rather than analytically) as “improved,” “unchanged,” “worse,” or “mixed.” This categorization was based on the results presented by the study authors without further interpretation of the results due to the nature of the review being undertaken. Due to updated scoping review methodology recommendations,^24^ a qualitative thematic analysis was not performed, which was a deviation from the scoping review protocol.^26^

### Role of the Funding Source

The Irish Society of Chartered Physiotherapists (ISCP) played no role in the design, conduct, or reporting of this review.

## Results

**Figure 1:**
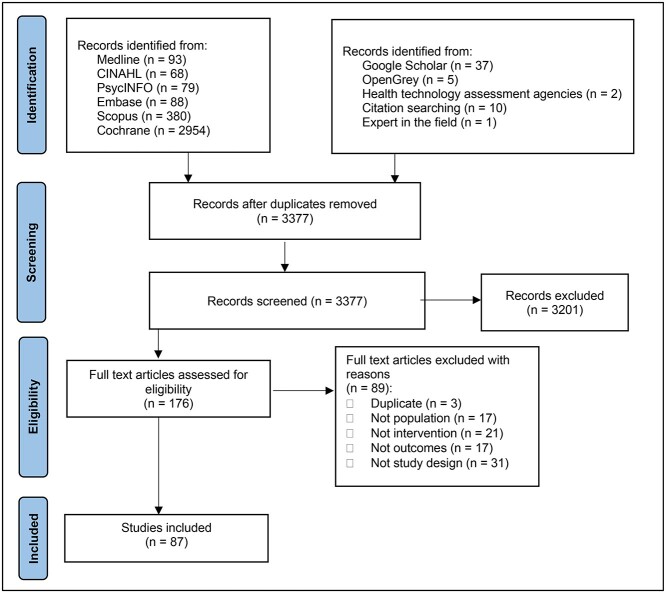
PRISMA flow diagram.

As presented in the PRISMA flow diagram (Fig. 1),^25^ from 3377 unique citations, 87 full-text studies were included.

### Description of Studies

A summary of the 87 studies included in this review is provided in Table 1 and Supplementary Table 2 (categorized by eHealth modality).

The most common study designs were randomized controlled trials (n = 60; 69%). Nonrandomized controlled trials consisted of observational (n = 10; 12%), qualitative (n = 8; 9%), mixed methods (n = 7; 8%), and randomized non-inferiority trials (n = 2; 2%). The majority of qualitative and mixed methods studies (n = 12; 80%) utilized interviews for data collection.

The included studies were conducted in 15 countries, including the United States (n = 28; 32%), Australia (n = 16; 18%), and Sweden (n = 11; 13%). Most studies were published in the last 5 years (n = 48; 55%). The mean age range of participants reported across studies was 27.3 to 75 years. Only 3 studies had participants with a mean age greater than 65 years.^30–32^

### eHealth Modality Type

Twenty-four studies reported the same eHealth modality: hence, 63 interventions were outlined (Tab. 2). The most common eHealth modality type was internet based (n = 22; 35%), with the least common single modality being virtual reality (n = 1; 2%). Almost one-fifth of interventions identified (n = 12; 19%) utilizing more than 1 eHealth modality, with the most common combination being internet based with telephone support (n = 8; 13%). Most interventions utilized a combination of face-to-face (ie, in-person) and eHealth modality format (n = 35; 56%).

**Table 2:** Overall Characteristics of eHealth-mediated Self-management Support Interventions^a^

**Variable**	**Intervention Count (n = 63)**
eHealth-modality type	
Internet based	22
Telephone based	15
Mobile phone application	8
Internet based with telephone support	8
Interactive voice response	3
Video teleconferencing	2
Mobile phone application with telephone support	1
Interactive voice response with telephone support	1
Internet based with video conferencing	1
Interactive voice response with telephone support and internet based	1
Virtual reality	1
Format	
Mixture of face-to-face and eHealth	35
eHealth only	28
Components of the interventions mapped to PRISMS taxonomy	
Adherence support and lifestyle interventions	59
Information provision and patient education	58
Remote monitoring with feedback and action plans	53
Training and rehearsal of psychological strategies	41
eHealth-facilitated clinical review	42
Interventions that contained all 5 PRISMS taxonomy components	23
Interventions contained ≤2 PRISMS taxonomy components	2
Type of provider	
Multiple	17
Psychologist/graduate psychology students	12
N/A, eHealth modality fully automated	12
Other*^b^*	10
Physical therapist	9
Nurse	2
Health coach	1
Location of intervention	
Multiple	34
Individual’s home	28
Outpatient	1
Frequency	
Mixed	29
Weekly	18
More than once per week	5
Daily	5
Unstructured, degree of frequency not specified	3
Monthly	2
More than once per day	1
Duration	
6–8 wk	18
9–20 wk	16
6 mo–1 y	14
3 wk–5 wk	7
Mixed	5
2 y	1
One-time access	1
Unspecified	1
Follow-up	
None	29
1–5 mo	13
11–12 mo	11
6–9 mo	10
Intervention development	
Use of framework/theory	48
Co-designed with individual	14

*^a^* N/A = not applicable; PRISMS = Practical Review in Self-Management Support.

*^b^* Indicates other providers, that is, health counselor, facilitator, psychiatrist, physical therapist, research staff, exercise physiologist, orthopedic specialist, and/or physician.

### Musculoskeletal Diagnosis

There were 5 groups of musculoskeletal diagnoses identified. The most frequent diagnosis was widespread musculoskeletal conditions (n = 41; 47%) (Tab. 1) followed by back pain (n = 27; 31%), with neck pain (n = 2; 2%) as the least common diagnosis. Most studies related to chronic or persistent presentations (n = 79; 91%).

### Study Outcomes and Results

Characteristics of the quantitative study outcomes are presented in Table 3. Sixty-seven studies (45%) assessed outcomes relating to body functions (ie, pain, anxiety, depression, etc), and 65 studies (44%) assessed outcomes relating to activities and participation (ie, function). The least common outcomes reported related to environmental factors such as medication and health care utilization (n = 17; 20%). The overall study outcomes were reported solely based on the findings reported by the study authors’ findings and were not subjected to any form of critical analysis. These results were categorized descriptively as “improved,” “unchanged,” or “worse.” When studies reported a combination of improved, unchanged, and/or worse outcomes, they were categorized as “mixed” results. Based on this method of categorization, 149 outcomes were reported, of which 50% (n = 75) improved, 1% (n = 2) worsened, 21% (n = 30) had no change, and 28% (n = 42) had mixed results. Of the 75 outcome categories that were improved, 30 (40%) were related to body functions, 38 (51%) were related to activities and participation, and 7 (9%) were related to environmental factors.

**Table 3:** Summary of Quantitative Study Outcomes^a^

**Common Outcomes**	**Description**	**No. of Studies**	**No. of Studies in Which Outcome Improved**
Body functions	Pain intensity, pain interference, anxiety, depression, sleep, self-efficacy	67	30
Activities and participation	Stress management, walking, function, employment	65	38
Environmental factors	Medication, health care utilization	17	7

*^a^* Based on the International Classification of Functioning, Disability and Health framework.

Supplementary Table 2 includes the findings from 8 qualitative and 7 mixed methods studies. The majority of these studies (n = 12; 80%) utilized interviews for data collection. It is difficult to summarize findings into meaningful categories given the variety of eHealth-mediated self-management support interventions. Two studies reported good satisfaction,^33^^,^^34^ 3 studies reported good acceptability,^35–37^ and 2 studies reported that participants with MSDs found the eHealth-mediated self-management support interventions to be beneficial.^38^^,^^39^

### eHealth-Mediated Self-Management Support Intervention Characteristics

eHealth modalities were overseen by a variety of providers (Tab. 2). The most common category was “multiple providers” (n = 17; 27%). The most common identifiable group of providers was psychologists or graduate psychology students (n = 12; 19%), with the same number of interventions fully automated with no provider involvement (n = 12; 19%). The least common was health coach (n = 1; 2%). The majority of interventions involved mixed frequencies ranging from 3 times per day to monthly (n = 29; 46%), with the least common frequency reported was more than once per day (n = 1; 2%). The majority of interventions lasted between 6 and 8 weeks (n = 18; 29%), whereas the least common durations reported included one-time access, 2 years, and unspecified (n = 1; 2% each). Almost one-half of the included studies (n = 29; 46%) reported posttreatment data only. The majority of eHealth-mediated self-management support interventions involved multiple locations such as a combination of outpatient with persons’ home (n = 34; 54%), with the outpatient setting the least common location reported (n = 1; 2%).

Adherence support and lifestyle interventions were the most common components of the PRISMS taxonomy identified (n = 59; 94%), whereas training and rehearsal of psychological strategies was the least common (n = 41; 65%). Most of the eHealth-mediated self-management support interventions were complex interventions with multiple components; only 2 studies (3%) contained 2 or less PRISMS taxonomy components.^40^^,^^41^

With respect to intervention development, 76% (n = 48) indicated the use of theories or frameworks. These included cognitive behavioral theory, acceptance and commitment theory, social cognitive theory, and the transtheoretical model of behavior change. Almost one-quarter of interventions (n = 14; 22%) involved individuals in the design of the intervention.

An overview of identified eHealth modality types with corresponding musculoskeletal diagnosis, outcomes, and intervention characteristics is provided in Table 4. The majority of studies evaluating internet-based interventions have been conducted in those with widespread musculoskeletal symptoms (n = 19; 63%), whereas the majority of telephone-based interventions have been assessed in populations with back pain (n = 9; 45%) and osteoarthritis (n = 8; 40%). Of the 10 studies included in this review relating to mobile phone applications, 6 (60%) were evaluated in those with back pain.

**Table 4:** Tabular Presentation of Scoping Review Outcomes^a^

**eHealth Modality Type (Intervention Count = 63)**	**Internet Based (n = 22)**	**Telephone Based (n = 15)**	**Modality + Telephone Support (n = 11)**	**Mobile Phone Application (n = 8)**	**IVR (n = 3)**	**Video Teleconferencing (n = 2)**	**VR (n = 1)**	**Internet Based With Video Conferencing (n = 1)**
MSK diagnosis (n = 87)	MSK conditions (n = 19)	MSK conditions (n = 1)	MSK conditions (n = 13)	MSK conditions (n = 2)	MSK conditions (n = 3)	MSK conditions (n = 1)	MSK conditions (n = 1)	
	Back pain (n = 7)	Back pain (n = 9)	Back pain (n = 5)	Back pain (n = 6)	Back pain (n = 1)			
	OA (n = 4)	OA (n = 8)						
		Knee pain (n = 1)		Knee pain (n = 1)		Knee pain (n = 1)		Knee pain (n = 2)
		Neck pain (n = 1)		Neck pain (n = 1)				
Quantitative study outcomes (n = 149)	Body functions (n = 23)	Body functions (n = 13)	Body functions (n = 15)	Body functions (n = 9)	Body functions (n = 3)	Body functions (n = 2)	Body functions (n = 1)	Body functions (n = 1)
	Activities and participation (n = 22)	Activities and participation (n = 15)	Activities and participation (n = 13)	Activities and participation (n = 8)	Activities and participation (n = 3)	Activities and participation (n = 2)	Activities and participation (n = 1)	Activities and participation (n = 1)
	Environmental factors (n = 4)	Environmental factors (n = 5)	Environmental factors (n = 4)	Environmental factors (n = 2)	Environmental factors (n = 1)			Environmental factors (n = 1)
Intervention provider (n = 63)	N/A, eHealth modality fully automated (n = 9)			N/A, eHealth modality fully automated (n = 2)			N/A, eHealth modality fully automated (n = 1)	
	Multiple (n = 5)	Multiple (n = 7)	Multiple (n = 2)	Multiple (n = 2)	Multiple (n = 1)			
	Psychologist/ graduate psychology students (5)		Psychologist/graduate psychology students (6)			Psychologist/ graduate psychology students (1)		
	Nurse (n = 1)	Nurse (n = 1)						
	Physical therapist (n = 1)	Physical therapist (n = 3)	Physical therapist (n = 1)	Physical therapist (n = 2)		Physical therapist (n = 1)		Physical therapist (n = 1)
	Other (n = 1)	Other (n = 4)	Other (n = 1); health coach (n = 1)	Other (n = 2)	Other (n = 2)			
Duration (n = 63)	3–5 wk (n = 5)			3 wk–5 wk (n = 2)			3 wk–5 wk (n = 1)	
	6–8 wk (n = 6)	6–8 wk (n = 2)	6–8 wk (n = 6)	6–8 wk (n = 2)		6–8 wk (n = 1)		
	9–20 wk (n = 6)	9–20 wk (n = 1)	9–20 wk (n = 2)	9–20 wk (n = 3)	9–20 wk (n = 1)	9–20 wk (n = 1)		9–20 wk (n = 1)
	6 mo–1 y (n = 2)	6 mo–1 y (n = 7)	6 mo −1 y (n = 3)					
	Mixed (n = 2)	Mixed (n = 3)			Mixed (n = 2)			
	One time access, unspecified (n = 1)	Unspecified (n = 1)		Unspecified (n = 1)				
		2 y (n = 1)						
Frequency	Daily (n = 1)	Mixed (n = 8)	Mixed (n = 11)	Mixed (n = 8)	Mixed (n = 2)	Mixed (n = 3)		Mixed (n = 1)
	Daily (n = 1)			Daily (n = 2)			Daily (n = 1)	
	Weekly (n = 9)		Weekly (n = 3)			Weekly (n = 2)		
	More than once per day (n = 1)							
	More than once/wk (n = 2)	More than once/wk (n = 1)		More than once/wk (n = 2)				
		Monthly (n = 2)						
	Unstructured (n = 1)	Unstructured (n = 1)		Unstructured (n = 2)				
Outcomes improved, worse, unchanged or mixed	Body functions: Mixed (n = 15) Improved (n = 5) Unchanged (n = 3)	Body functions: Mixed (n = 5) Improved (n = 5) Unchanged (n = 3)	Body functions: Mixed (n = 6) Improved (n = 8) Unchanged (n = 1)	Body functions: Mixed (n = 2) Improved (n = 7)	Body functions: Improved (n = 3)	Body functions: Improved (n = 1) Mixed (n = 1)	Body functions: Mixed (n = 1)	Body functions: Improved (n = 1)
	Activities and participation Improved (n = 9) Unchanged (n = 9) Mixed (n = 3) Worse (n = 1)	Activities and participation Improved (n = 9) Unchanged (n = 2) Mixed (n = 4)	Activities and participation Improved (n = 8) Unchanged (n = 4) Mixed (n = 1)	Activities and participation Improved (n = 6) Unchanged (n = 1) Mixed (n = 1)	Activities and participation Improved (n = 3)	Activities and participation Improved (n = 1) Mixed (n = 1)	Activities and participation Improved (n = 1)	Activities and participation Improved (n = 1)
	Environmental factors Improved (n = 2) Unchanged (n = 1) Mixed (n = 1)	Environmental factors Improved (n = 1) Unchanged (n = 3) Worse (n = 1)	Environmental factors Improved (n = 2) Mixed (n = 1) Unchanged (n = 1)	Environmental factors Improved (n = 2)	Environmental factors Improved (n = 1)			Environmental factors Unchanged (n = 1)

*^a^* IVR = interactive voice response; MSK = musculoskeletal; N/A = not applicable; OA = osteoarthritis; VR = virtual reality.

## Discussion

This scoping review mapped the available evidence on eHealth-mediated self-management support interventions for those with MSDs with regards to eHealth modalities, musculoskeletal diagnosis, and outcomes. To our knowledge, this is the first review comprehensively charting 7 different eHealth modalities and their various combinations with respect to self-management support. In total, 87 studies of considerable variation in eHealth modality, outcomes assessed, and results obtained fulfilled the inclusion criteria. eHealth-mediated self-management support interventions are an emerging area of practice in musculoskeletal disease, with most of the studies published in the last 5 years. COVID-19 may accelerate this further,^8^ with reported increases in eHealth use in Switzerland and Australia as a result of the pandemic ranging from 44.6% to 60%.^42^^,^^43^ However, these figures contrast sharply with findings reported by Werneke et al,^44^ indicating an overall eHealth usage rate of 6%. Furthermore, despite increased use of eHealth reported in certain settings, physical therapists may not plan to continue to provide eHealth modalities after the pandemic.^43^

Internet-based interventions were the most common eHealth modality. In keeping with previous reviews,^7^^,^^45^ the most common intervention delivery format was a combination of eHealth and face-to-face. It should be noted that during the COVID-19 pandemic, allied health clinicians managing those with MSDs rapidly introduced video teleconferencing and telephone into their clinical practice,^42^^,^^43^ not internet-based interventions. This is likely related to the fact that more people have access to smartphones than desktop personal computers,^46^ and not all internet-based interventions are accessible via smartphones.^47^ Other reasons may be the cost of developing and implementing internet-based self-management support interventions from an organizational perspective, along with issues around reimbursement that health care services across many countries receive for eHealth interventions.^48^ In response to the COVID-19 pandemic, advocacy from professional bodies supported the reimbursement of telephone and video teleconferencing services by numerous funding bodies,^49^^,^^50^ which paved the way for the recent rapid introduction of these eHealth modalities into practice in some settings.^42^^,^^43^ Although this move was necessary during the pandemic for many health care providers because it was the only option for individuals to receive musculoskeletal health care,^6^ it is worth noting that video teleconferencing was identified in only 2 included studies^32^^,^^51^ and hence requires further investigation. The first-hand experience of video teleconferencing and telephone that many clinicians and individuals with MSDs now have will certainly shift views, either positively or negatively.^6^ Given the lack of user (ie, individuals with MSDs) involvement in eHealth intervention design noted within this review and criticized repeatedly in the literature,^52^^,^^53^ it is of paramount importance that future research adopts a co-design model involving various stakeholders (eg, individuals with MSDs, clinicians, software developers, researchers) to ensure the success and long-term sustainably of eHealth as a method for service delivery.^54^

Almost one-half of the included studies (n = 41; 47%) involved participants with widespread musculoskeletal symptoms such as neck and back pain^55^ or fibromyalgia^56–58^ or chronic pain.^51^^,^^59–82^ This is not surprising given that research indicates that most people reporting musculoskeletal pain describe multisite pain, with single-site pain relatively rare.^83^ Furthermore, the more widespread the symptoms, the greater the impact on health and functioning.^83^ An important point to highlight is that telephone and video teleconferencing consultations were being utilized during the pandemic to manage a myriad of MSDs such as tendinopathy and joint and muscle strains despite very limited evidence of efficacy to support eHealth-mediated self-management support for these presentations^42^; the majority of the telephone-based studies identified within this review pertained to those with back pain and osteoarthritis. More recently, a retrospective observational design study published by Werneke et al^44^ reported that telehealth was administered equally across numerous musculoskeletal regions, with unadjusted physical function change similar for those using and no using telehealth. However, the authors urge caution in the interpretation of physical function change due to the study design. This, together with the gap identified in this scoping review, highlights the urgent need for high-quality research to develop and evaluate eHealth-mediated self-management support interventions for the broad range of MSDs managed by health care systems to provide better guidance for clinical practice.^42^ Only 5% of included studies involved individuals with an acute presentation (<12 weeks), which is similar to other reviews^84^^,^^85^ on self-management interventions. As a result, the effectiveness of self-management support interventions in this cohort remains unknown despite the importance of early self-management in those with MSDs.^86^ This gap within the literature may be partly related to the nature of MSDs, with recent-onset low back pain, for example, resolving quickly,^87^ often with minimal intervention.^88^ However, approximately 26% do not recover within 3 to 6 months,^89^ with research indicating that treatments are rarely effective at returning them to a pain-free life.^90^ It is likely that any eHealth-mediated self-management support intervention for those with acute MSDs would require different advice and support from that offered to those with chronic MSDs, in line with clinical guidelines.^86^

Although research indicates that self-management support has a positive impact on health care utilization in conditions such as asthma^23^ and cardiovascular disorders,^91^ there is a paucity of literature available on this outcome regarding MSDs. The eHealth-mediated self-management support intervention duration was relatively short despite improved treatment durability considered 1 potential advantage of eHealth modalities. Another limitation of the eHealth-mediated self-management support interventions identified within this scoping review is the lack of follow-up, with almost one-half of studies reporting posttreatment data only, a finding replicated in a review by Garg et al.^92^

The outcomes of the interventions used in the studies included in the review were descriptively categorized as improved, unchanged, or worse based solely on the study authors’ conclusions. The strength of the evidence underpinning the authors’ findings was not subjected to critical analysis, and, as a result, conclusions regarding the effectiveness of eHealth-mediated self-management support interventions for those with MSDs cannot be drawn. The aim of this scoping review was to review the breadth of the current literature and present the gaps that exist with scoping review methodology, not the most valid approach to evaluate effectiveness.^93^

### Study Limitations

Several limitations pertaining to this scoping review need to be acknowledged. This review includes English language papers only, and hence, potentially relevant evidence from other language may have been overlooked. Materials from sources such as guidelines, websites, or book chapters were not included. This may have introduced potential bias into the results. Finally, scoping reviews are inherently limited because the focus is to provide breath rather than depth of evidence on a particular topic.^24^ Hence, conclusions cannot be drawn regarding the most effective eHealth-mediated self-management support intervention for those with MSDs. However, this method was appropriate given the variety of options that eHealth includes and the varied nature of self-management interventions.

### Future Implications

Accessibility and sustainability are key challenges facing musculoskeletal health care services, challenges that have been further exacerbated by COVID-19. Robust evidence to support eHealth-mediated self-management is critical if these interventions are to be successfully adopted post COVID-19. At present, it is challenging to synthesize and interpret eHealth-mediated self-management support interventions given the different methodological approaches and outcome measurements utilized among the included studies. To ameliorate this, researchers should adopt standard eHealth reporting guidelines.^94^ This scoping review identified 2 studies evaluating the role of video teleconferencing in supporting self-management, an eHealth modality that was rapidly introduced into routine clinical practice in response to the COVID-19 pandemic. It is imperative that future research adopts a co-design model when evaluating the role of video teleconferencing and other eHealth modalities in supporting self-management. The majority of eHealth modalities were evaluated in populations with chronic pain, back pain, and osteoarthritis. To provide better guidance for clinical practice, high-quality research is also required for the broad range of MSDs managed by health care systems. Lastly, future studies should also evaluate the impact of these interventions on health care utilization^92^ and increase the length of follow-up.

## Supplementary Material

PTJ-2021-0509_R3_Supplemental_File_1_pzab307

PTJ-2021-0509_R3_Supplemental_Table_1_pzab307

PTJ-2021-0590_R3_Supplemental_Table_2_pzab307

## Systematic Review Registration

The protocol was registered with the Open Science Framework (https://osf.io/29rd6) and published.^26^
