# Supplementary material for: eHealth Interventions to Support Self-Management in People With Musculoskeletal Disorders, “eHealth: It’s TIME”—A Scoping Review
Source: Phys Ther. 2022 Jan 13;102(4):pzab307. doi: 10.1093/ptj/pzab307 (PMC8994513; doi:10.1093/ptj/pzab307)
Supplement: PTJ-2021-0509_R3_Supplemental_File_1_pzab307 [file ptj-2021-0509_r3_supplemental_file_1_pzab307.pdf]

eHealth modality definitions/explanations

Telephone supported

These interventions typically involve telephone support from health care professionals with expertise in the specific subject matter to supplement a structured internet intervention. The main aim is to 'provide support and encouragement for use of the internet intervention, and to address any concerns in relation to the internet-based content'.[1]

Telephone-based

Intervention delivered via telephone calls between a patient and health care professional (2-way interaction) to support self-management[2] through knowledge exchange, goal setting, action planning and maintenance.[3]

Internet-based

Structured programs that participants can interact with by utilizing mobile devices or computers. They can 'support self-management by facilitating goal setting, self-monitoring and providing behavioral/symptom-related feedback'.[1(p. 3)]

Virtual reality

A three-dimensional computer generated environment, which facilitates an individual to interact with, explore and manipulate objects by stimulating human senses.[4]

Interactive voice response

This technology is a 'method for interaction between an individual and a computer through the medium of a telephone'[(p. 39)] using the touch-tone keypad.[5] Typically an automated script poses questions and the caller keys in responses using the telephone keypad.

Mobile phone applications

Mobile-based or mobile-enhanced programs that provide health-related services for tablets, smartphones and other communication devices.

Video teleconferencing

This involves a combination of high-quality audio and video via internet protocol networks to enable real-time interactions between individuals which can be utilized for clinical support, healthcare management and diagnostic purposes.

References

1. Geraghty AW, Stanford R, Little P, et al. Using an internet intervention to support self-management of low back pain in primary care: protocol for a randomised controlled feasibility trial (SupportBack). *BMJ open.* 2015;5(9):e009524.
2. Bennell KL, Egerton T, Bills C, et al. Addition of telephone coaching to a physiotherapist-delivered physical activity program in people with knee osteoarthritis: a randomised controlled trial protocol. *BMC Musculoskelet Disord.* 2012;13:246.
3. Gale J, Skouteris H. Health coaching: facilitating health behavior change for chronic condition prevention and self-management. *Applied topics in health psychology*: Wiley-Blackwell; 2013:15-28.
4. Riva G. From Telehealth to E-health: Internet and distributed virtual reality in health care. *Cyberpsychol Behav.* 2000;3(6):989-998.
5. Naylor MR, Helzer JE, Naud S, Keefe FJ. Automated telephone as an adjunct for the treatment of chronic pain: a pilot study. *J Pain.* Dec 2002;3(6):429-438.
