# Supplementary material for: eHealth Interventions to Support Self-Management in People With Musculoskeletal Disorders, “eHealth: It’s TIME”—A Scoping Review
Source: Phys Ther. 2022 Jan 13;102(4):pzab307. doi: 10.1093/ptj/pzab307 (PMC8994513; doi:10.1093/ptj/pzab307)
Supplement: PTJ-2021-0509_R3_Supplemental_Table_1_pzab307 [file ptj-2021-0509_r3_supplemental_table_1_pzab307.pdf]

Inclusion and Exclusion Criteria

Inclusion Criteria	Exclusion Criteria
Participants:<br>• Adults with a MSD	Participants:<br>• With pain of specific pathological origin (eg, infection etc)<br>• Following surgery<br>• Pregnancy
Concept:<br>• eHealth interventions[a] that support self-management[b]	Concept:<br>• eHealth modalities that involve remote monitoring without an explicit decision-making role
Context:<br>• All health system models<br>• All countries.	Context<br>• N/A
Types of studies:<br>• All qualitative & quantitative dominant studies<br>• All mixed method studies<br>• Published in English	Types of studies:<br>• Study protocols<br>• Dissertations, theses, conference abstracts & case studies<br>• All sources without reference to empirical research

[a] An eHealth modality is considered to be some specific form of technology that is applied in the context of health care.[4]

[b] Self-management was defined as any intervention that aimed to empower patients to be active decision makers who deal with social, emotional or medical management of their condition with the aim of improving their independence and quality of life.[9]
