# Supplementary material for: eHealth Interventions to Support Self-Management in People With Musculoskeletal Disorders, “eHealth: It’s TIME”—A Scoping Review
Source: Phys Ther. 2022 Jan 13;102(4):pzab307. doi: 10.1093/ptj/pzab307 (PMC8994513; doi:10.1093/ptj/pzab307)
Supplement: PTJ-2021-0590_R3_Supplemental_Table_2_pzab307 [file ptj-2021-0590_r3_supplemental_table_2_pzab307.pdf]

‘eHealth: It’s TIME’

Table 2. Characteristics of Included Studies (n = 87)

eHealth Modality Type	Study & Year (Reference)<br><br>Design	Target Population<br><br>Study Size<br>Age (y)	Format<br><br>Provider<br><br>Frequency<br><br>Duration<br><br>Follow-up	Description of Intervention	Components of the Interventions Mapped to PRISMS Taxonomy	Reported Study Outcomes /Author’s Findings	Study Results
Internet-based (n=22)							
Internet-based	Chiauzzi et al 2010[95]<br><br>RCT	Chronic back pain<br><br>N = 209 (E = 104, C = 105)<br><br>Age = 46.14	eHealth only<br><br>Provider: N/A - eHealth modality fully automated<br><br>Frequency: twice per week<br><br>Duration: four weeks<br><br>Follow-up: six months	painACTION-Back Pain internet program<br><br>• Lessons, interactive tools, personalized assessments, & articles based on CBT & self-management principles<br><br>*Comparator: text-based material*	• Information provision & patient education<br>• Training & rehearsal of psychological strategies<br>• Adherence support & lifestyle interventions	Body Functions:<br>• Improved coping, pain, anxiety & depression<br>• Mean self-efficacy change did not differ between groups<br><br>Activities & Participation:<br>• Improved stress<br>• Physical functioning did not differ between groups	
Internet-based	Williams et al (2010)[57]<br><br>RCT	Fibromyalgia<br><br>N = 118 (E = 59, C = 59)<br><br>Age = 50.46	eHealth only<br><br>Provider: N/A - eHealth modality fully automated<br><br>Frequency of internet based self-management modules: Approx. weekly, recorded use of self-management skills monthly<br><br>Duration: Six months<br><br>Follow-up: none	Standard care plus access to a Web-Enhanced Behavioral Self-Management program - “Living Well with Fibromyalgia”<br><br>13 modules, each of which featured:<br>• Video lecture<br>• Written summaries of video lecture<br>• Homework & self-management forms<br>• Supplemental educational materials	• Training & rehearsal of psychological strategies<br>• Information provision & patient education<br>• Remote monitoring with feedback & action plans<br>• Adherence support & lifestyle interventions	Body Functions:<br>• Pain improved<br>• Fatigue, sleep, depression & anxiety did not differ between groups<br><br>Activities & Participation:<br>• Physical functioning improved	

				unique to each topic (e.g., audio relaxation exercises & readings)<br><br>*Comparator: standard care*			
Internet-based	Kristjánsdóttir et al (2011)[39]<br><br>Mixed methods	Chronic musculoskeletal pain<br><br>N = 6<br><br>Age = 36.3	Mixture of face-to-face & eHealth<br><br>Provider: nurse<br><br>Frequency: mixed; diary entries three times per day, feedback daily except weekends<br><br>Duration: 4 weeks<br><br>Follow-up: none	Internet based intervention via a web-enabled mobile phone<br>• One 1:1 face-to-face meeting with therapist – provided with written exercises, relaxation CD and lent web-enabled mobile phone<br>• Completed 3 diary entries per day – prompted by text message<br>• Therapist provided daily feedback (except weekends)	• Information provision & patient education<br>• Remote monitoring with feedback & action plans<br>• Adherence support & lifestyle interventions<br>• Training & rehearsal of psychological strategies<br>• eHealth-facilitated clinical review	• Supportive, meaningful and user friendly<br>• High response rate with few technical issues<br>• Feasible	
Internet-based	Jelin et al (2012)[96]<br><br>Qualitative	Chronic musculoskeletal pain<br><br>N= 7<br><br>Age = unavailable	See Kristjánsdóttir et al (2011)[39] for internet-based intervention.<br>This followed in-patient multidimensional rehabilitation	See Kristjánsdóttir et al (2011)[39] for internet-based intervention.<br>This followed in-patient multidimensional rehabilitation	See Kristjánsdóttir et al (2011)[39] for internet-based intervention.<br>This followed in-patient multidimensional rehabilitation	Mixed<br>• Experienced support via feedback & diaries<br>• However, ambivalence in:<br>• Using internet-based technology<br>• Feedback as challenging but positive<br>• Impersonal relationship with therapist	

Internet-based	Carpenter et al (2012)[97]<br><br>RCT	Chronic LBP<br><br>N = 141 (E = 70, C = 71)<br><br>Age = 42.5	eHealth only<br><br>Provider: N/A - eHealth modality fully automated<br><br>Frequency: weekly<br><br>Duration: three weeks<br><br>Follow-up: week 6 (both groups had used the Wellness Workbook)	Online CBT intervention – Wellness Workbook<br><br>6 chapters, 189 pages<br>• Didactic instruction – chapter topic & mindfulness<br>• Animation<br>• Audio-recorded patient stories<br>• Reflective exercises<br>• Interactive exercises<br>• Guided relaxation & mediation exercises<br><br>*Comparator: waiting list*	• Training & rehearsal of psychological strategies<br>• Information provision & patient education<br>• Adherence support & lifestyle interventions	Body Functions:<br>• Pain severity unchanged<br>• Self-efficacy & fear avoidance beliefs improved<br><br>Activities & Participation:<br>• Physical function improved	
Internet-based	Ruehlman et al (2012)[63]<br><br>RCT	Chronic Musculoskeletal conditions<br><br>N = 330 (E = 165, C = 165)<br><br>Age = 44.93	eHealth only<br><br>Provider: N/A - eHealth modality fully automated<br><br>Frequency: Twice/week<br><br>Duration: Eight weeks<br><br>Follow-up: Fourteen weeks	Internet based ‘Chronic Pain Management Program’<br>• Custom learning plan<br>• Interactive learning modules<br>• Goalistics Navigator<br>• Social networking<br><br>*Comparator: waiting list*	• Training & rehearsal of psychological strategies<br>• Adherence support & lifestyle interventions<br>• Information provision & patient education<br>• Remote monitoring with feedback & action plans	Body functions:<br>• Pain severity, pain-related interference, emotional functioning, catastrophizing & pain-related fear improved<br><br>Activities & Participation:<br>• Physical functioning improved	
Internet-based	Wilson et al (2015)[76]<br><br>RCT	Chronic musculoskeletal conditions<br><br>N = 114 (E = 57, C = 57)<br><br>Age = 49.4	See Ruehlman et al (2012)[63] for intervention<br><br>Follow-up: none	See Ruehlman et al (2012)[63]<br><br>*Comparator: wait list control*	See Ruehlman et al (2012)[63] f	Body functions:<br>• Improvement in pain self-efficacy in E post treatment<br>• Pain intensity unchanged, although increased program engagement associated with improvements	

						Activities & Participation:<br>• Pain related functional interference- unchanged post treatment<br><br>Environmental factors:<br>• Improvements in opioid misuse measures<br>• Health care utilization – unchanged	
Internet-based	Wilson et al (2017)[98]<br><br>Qualitative	Chronic musculoskeletal conditions, prescribed opioid medicines<br><br>N = 47<br><br>Age = 47	See Ruehlman et al (2012)[63]	See Ruehlman et al (2012)[63]	See Ruehlman et al (2012)[63]	Perceived program benefits:<br>• Positive reframing, improved accountability, & feeling supported<br><br>Perceived program challenges:<br>• Ease of use & desire for personalizing	
Internet-based	Bossen et al (2013)[99]<br><br>RCT	Knee &/or hip OA<br><br>N = 199 (E = 100, C = 99)<br><br>Age = 62	eHealth only<br><br>Provider: N/A - eHealth modality fully automated<br><br>Frequency: Once/week<br><br>Duration: Nine weeks<br><br>Follow-up: One year	Fully automated behavior graded activity program – ‘Join2move’<br>• Baseline test<br>• Goal setting<br>• Time contingent PA objectives<br>• Text messages to promote PA<br>• Information about OA, lifestyle & videos<br>• Automated e-mails	• Information provision & patient education<br>• Remote monitoring with feedback & action plans<br>• Adherence support & lifestyle interventions<br>•	Body functions:<br>• Pain self-efficacy improved at 3 months, not maintained at 12 months<br>• Active pain coping skills unchanged<br><br>Activities & Participation:<br>• Physical function improved at 3 months, not maintained at 12 months	

				Comparator: waiting list		• At 3 months, self-reported PA did not differ between groups<br>• At 12 months, improved subjective & objective PA	
Internet-based	Bossen et al (2013)[100]<br><br>Mixed methods	Knee &/or hip OA<br><br>N = 15 (10 users & 5 non-users)<br><br>Age = unavailable for this cohort	See Bossen et al (2013)[99]	See Bossen et al (2013)[99]	See Bossen et al (2013)[99]	• Participants felt that the automatic gradual increase of PA as well as working towards a short-term goal were mechanisms that supported them<br>• Compared with face-to-face treatments, the flexibility of completing modules at one’s own pace without time or travel restrictions was cited as a major advantage.<br>• Lack of human involvement was a disadvantage for older patients, those with a co-morbidity.	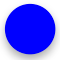

Internet-based	Buhrman et al (2013)[101]<br><br>RCT	Chronic Musculoskeletal conditions<br><br>N = 72 (E = 36, C = 36)<br><br>Age = 40.1	eHealth only<br><br>Provider: Graduate psychology students<br><br>Frequency: Weekly<br><br>Duration: Eight weeks<br><br>Follow-up: Six months	Guided internet-based CBT intervention<br>• 8 weekly modules – information part & practical part including assignments<br>• Homework submission via secure platform<br>• Written feedback from therapist via secure platform – encouragement, advice & non-specific support<br><br>*Comparator: moderated online discussion group*	• Information provision & patient education<br>• Remote monitoring with feedback & action plans<br>• eHealth-facilitated clinical review<br>• Training & rehearsal of psychological strategies<br>• Adherence support & lifestyle interventions	Body functions:<br>• Catastrophizing, pain-related distress, anxiety & depression improved<br>• Maintenance of improvement exhibited at 6-month follow-up<br><br>Activities & Participation:<br>• QOL unchanged	
Internet-based	Krein et al (2013)[102]<br><br>RCT	Chronic LBP<br><br>N = 229 (E = 111, C = 118)<br><br>Age = 51.6	Mixture of face-to-face & eHealth<br><br>Provider: Multiple providers – physical therapist delivered face-to-face back class, research staff participated in & monitored forum posts<br><br>Frequency of internet-based program: mixed – pain or activity related messages on website changed every second day, other components weekly<br>Frequency/duration of back class – not specified, had to attend prior to participation<br><br>Duration: Twelve months	Pedometer-based, internet-based intervention based on 'Stepping Up to Health' program<br><br>• Uploading pedometer<br>• Internet-based intervention - automated walking goals, feedback, motivational messages<br>• Social support via e-community<br>• Weekly email reminders to upload pedometer data<br><br>*Comparator: standard care with pedometer*	• Remote monitoring with feedback & action plans<br>• Adherence support & lifestyle interventions – e-communities, weekly email reminders<br>• Information provision & patient education<br>•	Body functions:<br>• Fear avoidance beliefs did not differ between groups at any time point<br>• Self-efficacy appeared to be the same or worse at 6 months for both groups; this difference did not persist at 12 months<br><br>Activities & Participation:<br>• Disability improved post-treatment, was not maintained at 12 months	

Internet-based	Kristjánsdóttir et al (2013)[70]<br><br>RCT	Chronic Musculoskeletal conditions<br><br>N = 140 (E = 70, C = 70)<br><br>Age = 44.2	Mixture of face-to-face & eHealth<br><br>Provider: Multiple – nurse for face-to-face session, inpatient rehab likely MDT & feedback by therapists<br><br>Frequency: Three diary entries per day<br><br>Duration: Four weeks<br><br>Follow-up: Five months & See Kristjánsdóttir et al (2013)[71] for 11 month follow-up data	• All participated in four-week inpatient MDT program<br>• All received access to non-interactive website with self-help pain management material<br>• One hour individual face-to-face session, lent a smartphone<br>• Internet-based ACT-based diaries via smartphone (16-24 questions)<br>• Personalised daily written ACT-based feedback from therapist<br>• Audio files with guided mindfulness exercises<br><br>*Comparator: standard care with access to non-interactive website with self-help pain management material*	• Information provision & patient education<br>• Remote monitoring with feedback & action plans<br>• eHealth-facilitated clinical review<br>• Adherence support & lifestyle interventions<br>• Training & rehearsal of psychological strategies	Body functions:<br>• Catastrophizing improved post treatment & at 5 months<br>• Pain unchanged<br>• Sleep improved improvement at 5-month follow-up<br><br>Activities & Participation:<br>• Physical functioning improved at 5-month follow-up	
Internet-based	Kristjánsdóttir et al (2013)[71]<br><br>RCT	Chronic Musculoskeletal conditions<br><br>N = 140 (E = 70, C = 70)<br><br>Age = 44.2	See Kristjánsdóttir et al (2013)[70] – 11 month follow-up	• See Kristjánsdóttir et al (2013)[70]	• See Kristjánsdóttir et al (2013)[70]	Body functions:<br>• Catastrophizing & pain levels comparable to C at 11-month follow-up<br><br>Activities & Participation:<br>• Physical functioning comparable to C at 11-month follow-up	

Internet-based	Nes et al (2017)[80]<br><br>RCT	Chronic musculoskeletal conditions<br><br>N = 48 from 2 arm RCT See Kristjánsdóttir et al (2013)[70]<br><br>Age = 43	See Kristjánsdóttir et al (2013)[70]	• See Kristjánsdóttir et al (2013)[70]	• See Kristjánsdóttir et al (2013)[70]	Body functions:<br>• Pain fear, avoidance & positive feelings improved<br><br>Activities & Participation:<br>• Performance of physical activities worsened over time	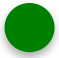<br>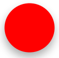
Internet-based	Ljótsson et al (2014)[56]<br><br>Obs	Fibromyalgia<br><br>N = 41<br>Age = 52	eHealth only<br><br>Provider: Graduate psychology students<br><br>Frequency: weekly<br><br>Duration: 10 weeks<br><br>Follow-up: 6 months	Internet-based ACT programme<br>• Treatment material divided into five successive steps<br>• Therapeutic contact via messages<br>• Access to online closed discussion forum	• Training & rehearsal of psychological strategies<br>• Information provision & patient education<br>• Remote monitoring with feedback & action plans<br>• Adherence support & lifestyle interventions<br>• eHealth-facilitated clinical review<br>•	Body functions:<br>• FM symptoms & impact, fatigue, depression & anxiety improved post treatment, with improvements maintained at 6-month follow-up.<br><br>Activities & Participation:<br>• Disability & QOL improved post treatment, with improvements maintained at 6-month follow-up<br><br>Environmental factors:<br>• Societal cost reductions /improvements that offset the treatment costs within 2 months of treatment completion	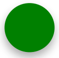<br>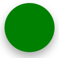<br>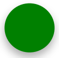

Internet-based	Riva et al (2014)[103]<br><br>RCT	Chronic LBP<br><br>N = 51 (E = 27, C = 24)<br><br>Age = 47.5	eHealth only<br><br>Provider: N/A - eHealth modality fully automated<br><br>Frequency: weekly<br><br>Duration: 8 weeks<br><br>Follow-up: none	Internet-based intervention with interactive features – 'ONESELF'<br>• Library<br>• Frequently asked questions<br>• First aid section<br>• Virtual gym<br>• Testimonials & Commentaries sections<br>• Weekly Action Plan<br>• Quiz Game<br>• Reminder short message service<br><br>*Comparator: internet-based intervention with no access to or knowledge of interactive sections*	• Information provision & patient education<br>• Adherence support & lifestyle interventions<br>• Remote monitoring with feedback & action plans<br>•	Body functions:<br>• Empowerment improved in E<br>• Pain burden improved, but to equal measures in both groups<br><br>Activities & Participation:<br>• Frequency of physical exercise improved but to equal measures in both groups<br><br>Environmental factors:<br>• Medication misuse improved	
Internet-based	Trompetter et al (2015)[75]<br><br>RCT	Chronic Musculoskeletal conditions<br><br>N = 238 (E = 82, internet based control Expressive Writing = 79, wait list control =77)<br><br>Age = 52.8	eHealth only<br><br>Provider: Graduate psychology students<br><br>Frequency: weekly<br><br>Duration: 9 – 12 weeks<br><br>Follow-up: 3 months	Internet based guided ACT self-management intervention – 'Living with Pain'<br>• 9 modules – with each module using text, experiential exercises & metaphors<br>• Mindfulness exercises<br>• ACT-participant stories<br>• Can keep personal diary<br>• Counseling support via email within encrypted system<br><br>Expressive writing control condition<br>• 9 modules on expressive writing	• Training & rehearsal of psychological strategies<br>• Information provision & patient education<br>• Adherence support & lifestyle interventions<br>• Remote monitoring with feedback & action plans<br>• eHealth-facilitated clinical review<br>•	Body functions:<br>• Pain interference improved post treatment & at 3 month follow-up compared to Expressive Writing control but not wait list control<br>• Improvements in depression, pain intensity & catastrophising compared to both other groups at 3 months follow-up<br><br>Activities & Participation:<br>• Disability improved	

				• Each module started with psycho-education, followed by a specific writing assignment<br>• Included online personal diary<br><br>*Other comparator: wait list control*		compared in E compared to both other groups at 3 month follow-up	
Internet-based	Irvine et al (2015)[104]<br><br>RCT	LBP within past 3 months<br><br>N = 597 (E = 199, alternate care group = 199, C = 199)<br><br>Age = unavailable	eHealth only<br><br>Provider: N/A - eHealth modality fully automated<br><br>Frequency: Mixed – tracked pain management activities daily, received weekly emails with messages & prompts<br><br>Duration: 8 weeks<br><br>Follow-up: 4 months (8 weeks after intervention concluded)	Mobile-Internet based intervention – ‘FitBack’<br><br>• Pain & activity self-monitoring tool<br>• Gain-framed text & video messages, which are segmented to address issues specific to each job type – sitters, standers, drivers & lifters<br>• Journaling feature<br>• Provides graphs to help users identify trends in pain level<br><br>Alternate care group:<br>• Initial email & 8 reminder emails, which included links to 6 websites re LBP<br><br>*Other comparator: waitlist control*	• Training & rehearsal of psychological strategies<br>• Adherence support & lifestyle interventions<br>• Information provision & patient education<br>• Remote monitoring with feedback & action plans	Body functions:<br>• Greater improvement in pain compared to C & alternate care group at 4-month follow-up<br>• Catastrophising was unchanged<br>• Self-efficacy improved<br><br>Activities & Participation:<br>• Improved functionality, quality of life, & well-being compared to C post treatment & at follow-up	
Internet-based	Rini et al (2015)[30]<br><br>RCT	Knee or hip OA<br><br>N = 113 (E = 58, C = 55)<br><br>Age = 67.6	Mixture of face-to-face & eHealth<br><br>Provider: other – research staff<br><br>Frequency: weekly<br><br>Duration: 8 weeks<br><br>Follow-up: none	Automated, Internet-based version of PCST called ‘PainCOACH’<br><br>• At baseline visit, motivational interviewing techniques<br>• 8 modules with no therapist contact<br>• Interactive training in cognitive or behavioral pain coping skills<br>• ‘Virtual’ coach	• Information provision & patient education<br>• Remote monitoring with feedback & action plans<br>• Adherence support & lifestyle interventions<br>• Training & rehearsal of psychological strategies	Body functions:<br>• Women in E demonstrated improvements in pain compared to C post intervention<br>• Self-efficacy improved in E (M = 12) compared to C post treatment	

				• Optional automated practice reminders<br>• Encouraging messages<br>• Badges earned by completing training modules etc.<br>• Self-monitoring - COACHtrack<br>• Coachchat – read & share experiences of PCST<br><br>*Comparator: standard care*		• Pain anxiety remained unchanged<br><br>Activities & Participation:<br>• Pain-related interference with functioning was unchanged	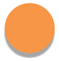
Internet-based	Nordin et al (2016) [77]<br><br>RCT	Chronic musculoskeletal conditions<br><br>N = 109 (E = 60, C = 49)<br><br>Age = 43	Mixture of face-to-face & eHealth<br><br>Provider: Multiple - MMR consisted of treatment from at least three different healthcare professionals (physiotherapist, physician, OT, psychologist, or psychosocial counsellor, nurse)<br><br>Frequency: Mixed – internet-based intervention – weekly for first 8 weeks<br>MMR – at least 2-3 sessions weekly for first 6 weeks<br><br>Duration: 4 months<br><br>Follow-up: 12 months	MMR + Web-BCPA<br><br>MMR:<br>• CB approach<br>• Supported by a rehabilitation coordinator<br>• Copy of individualised rehabilitation plan ± significant others<br>• Home exercise<br>• Education<br>• Relaxation<br>• Mindfulness<br>• Counselling<br>• PT may consist of acupuncture, manual therapy, TENS, hydrotherapy<br>• OTs may provide Ergonomics, activity planning, & functional training<br><br>Web-BCPA<br>• No therapist guidance<br>• 8 modules<br>• Information<br>• Interactive assignments (self-tests & self-developed action plans)<br>• Exercises, assimilated via educational texts, videos	• Information provision & patient education<br>• Remote monitoring with feedback & action plans<br>• Adherence support & lifestyle interventions	Body functions:<br>• Self-efficacy & pain intensity unchanged<br>• Catastrophizing improved at 4- & 12-month follow-up	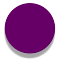

				• Writing tasks<br>• Relaxation & basic body awareness therapy exercises<br><br>*Comparator: MMR*			
Internet-based	Calner et al (2017)[78]<br><br>RCT	Chronic musculoskeletal conditions<br><br>N = 109 (E = 60, C = 49)<br><br>Age = 43	See Nordin et al 2016[77]	See Nordin et al 2016[77]	See Nordin et al 2016[77]	Body functions:<br>• Average pain intensity - unchanged<br><br>Activities & Participation:<br>• Pain related disability, work ability & health related QOL - unchanged	
Internet-based	Nordin et al (2017)[105]<br><br>Qualitative	Chronic musculoskeletal conditions<br><br>N = 19<br><br>Age = 45	See Nordin et al 2016[77]	See Nordin et al 2016[77]	See Nordin et al 2016[77]	• Increased self-confidence & empowerment in dialogue with healthcare professionals.<br>• Solitary work had an important role to acquire knowledge & insights<br>• Higher user control regarding timing may further increase participation	
Internet-based	Pearson et al (2016)[106]<br><br>Mixed methods	Widespread musculoskeletal conditions<br><br>(Hip, Knee, back pain or multiple)<br><br>N =83 for postal survey (42% response rate)<br>Age = 67	eHealth only – primarily codesign study<br><br>Provider: N/A - eHealth modality fully automated<br><br>Frequency: Unstructured – Degree of frequency not specified	Internet-based version of ESCAPE-pain<br>• Codesign study<br>• Protype website with limited function and offline material	• Information provision & patient education<br>• Remote monitoring with feedback & action plans<br>• Adherence support & lifestyle interventions<br>• Training & rehearsal of psychological strategies	• Older people more likely to accept internet-based program as an adjunct to face-to-face care rather than a replacement	

		Focus Groups (n = 24)<br>Age = unavailable<br><br>Interviews = 8<br>Age = unavailable	Duration: One time access<br><br>Follow-up: none			• Have concerns about safe use & quality of information<br>• Users require a credible website that provides personalized information, support, monitoring, & feedback.	
Internet-based	Johnson et al (2017)[82]<br><br>Obs	Chronic musculoskeletal conditions<br><br>N = 69<br><br>Age = 50.3	eHealth only<br><br>Provider: N/A – eHealth only<br><br>Frequency: daily<br><br>Duration: 30 days<br><br>Follow-up: none	Internet based pain self-management program 'Health eRide: Your Journey to Managing Pain'<br><br>• Can select avatar to represent themselves<br>• Assessments of pain<br>• Stage of change for pain self-management with feedback & 'report card' on same<br>• Personal activity centre – 56 interactive activities designed to activate change process<br>• Text messages<br>• Health eRide facebook page	• Information provision & patient education<br>• Remote monitoring with feedback & action plans<br>• Adherence support & lifestyle interventions<br>• Training & rehearsal of psychological strategies	Body functions:<br>• Pain & pain impact – improved<br><br>Activities & Participation:<br>• Stress management – improved	
Internet-based	Lin et al (2017)[79]<br><br>RCT	Chronic musculoskeletal conditions<br><br>N= 302 (with guidance = 100, without guidance = 101, C = 101)<br><br>Age = 51.7	eHealth only<br><br>Provider: Clinical psychologists<br><br>Frequency: weekly<br><br>Duration: 8 weeks<br><br>Follow-up: 6 months	Internet-based ACT intervention – 'ACTonPain"<br><br>• Introduction & 7 modules<br>• Automated text messages<br><br>With guidance<br>• Personalized & standardized feedback via email<br><br>Without guidance<br>• No feedback	• Information provision & patient education<br>• Training & rehearsal of psychological strategies<br>• Adherence support & lifestyle interventions<br>• Remote monitoring with feedback & action plans<br>• eHealth-facilitated clinical review	Body functions:<br>• Improved depression in unguided group compared to C at 6 months<br>• Improved pain interference in guided group compared to C post treatment & at 6 months.<br>• No differences between guided & unguided	

				Other comparator: waitlist control		groups with regards to effectiveness<br>• No differences in pain intensity or anxiety<br><br>Activities & Participation:<br>• No differences in mental or physical QOL	
Internet-based	Bendelin et al (2018)[107]<br><br>Qualitative	Chronic musculoskeletal conditions<br><br>N = 29<br><br>Age = 37	Mixture of face-to-face & eHealth – aftercare following inpatient rehab<br><br>Provider: multiple – pain psychologist, could consult with OT, PT and pain physician<br><br>Frequency: weekly<br><br>Duration: 20 weeks<br><br>Follow-up: none	Internet-based aftercare programme (ACP)<br>• Resembled self-help book with 8 modules<br>• Assignments and worksheets<br>• Extensive therapist guidance during first 4 weeks, worked more independently during latter 4 modules	• Information provision & patient education<br>• Training & rehearsal of psychological strategies<br>• Adherence support & lifestyle interventions<br>• Remote monitoring with feedback & action plans<br>• eHealth-facilitated clinical review	• Value-based goals are important<br>• Experiencing a beneficial effect of acceptance strategies can facilitate with problem solving & commitment to treatment<br>• Research needed to investigate whether individualized therapist guidance is helpful to establish goals & contribute to maintenance	
Internet-based	Bennell et al (2018)[108]<br><br>RCT	Osteoarthritis<br><br>(Hip OA)<br><br>N = 144 (E = 73, C = 71)<br><br>Age = 61.3	Mixture of face-to-face & eHealth<br><br>Provider: physiotherapist<br><br>Frequency: mixed. Online education & internet based intervention – weekly, physiotherapy – every 3 weeks, HEP three times per week	Automated internet based PCST prior to HEP<br><br>First 8 weeks<br>• Online education – 8 information sheets, one per week<br>• PCST – 'PainCoach' as per Rina et al (2015)[30]<br><br>Weeks 8 -24	• Information provision & patient education<br>• Training & rehearsal of psychological strategies<br>• Remote monitoring with feedback & action plans<br>• Adherence support & lifestyle interventions	Body functions:<br>• Pain coping improved at week 8 & sustained at 24 & 52 weeks in E<br>• Hip pain on walking improved in both E & C, no difference between groups	

			Duration: mixed. Online education & internet based intervention – 8 weeks, physiotherapy – weeks 8 – 24 (16 weeks), HEP from weeks 8 – 52 – 44 weeks in total<br><br>Follow-up: 12 months	• 5 physiotherapy sessions face-to-face for HEP (30 mins in duration)<br><br>*Comparator: online* education only in first 8 weeks – 8 information sheets, one per week<br><br>*Weeks 8 -24*<br>*5 physiotherapy sessions face-to-face for HEP (30 mins in duration)*		Activities & Participation:<br>• Greater improvement in function at week 8 in E compared to C<br>• Groups did not differ at week 24<br><br>Environment factors:<br>• Medication use was similar in both groups over the study period	
Internet-based	Petrozzi et al (2019)[40]<br><br>RCT	Chronic LBP<br><br>N = 108 (E = 54, C = 54)<br><br>Age = 50.4	Mixture of eHealth & face to face<br><br>Provider: multiple - physiotherapy or chiropractor<br><br>Frequency: mixed. Standard physical treatment – 12 sessions over 8 weeks. Internet based intervention - weekly<br><br>Duration: mixed. Standard physical treatment – 8 weeks. Internet based intervention – 5 weeks.<br><br>Follow-up:6 & 12 month	Internet-based psychological program (MoodGYM) in addition to standard physical treatment<br>• Weekly telephone reminder – MoodGYM – research assistant<br>• If report distress as a result of MoodGYM – referral to psychologist<br><br>*Comparator: 12* standard physical treatment sessions	• Training & rehearsal of psychological strategies	Body functions:<br>• Self-efficacy – did not differ between groups at 6 or 12 months<br>• No additional benefit to MoodGYM with standard physical treatment<br><br>Activities & Participation:<br>• Disability – did not differ between groups at 6 or 12 months<br>• No additional benefit to MoodGYM with standard physical treatment	

Internet-based	Schlicker et al (2020)[109]<br><br>RCT	Chronic back pain with recurrent major depressive disorder<br><br>N = 76 (E = 40, C = 36)<br><br>Age = 50.7	eHealth only<br><br>Provider: Psychologist<br><br>Frequency: mixed - module & feedback – weekly. Could avail of text message coach also.<br><br>Duration: 9 weeks with booster 4 weeks after – in total, 13 weeks<br><br>Follow-up: 6 months	Internet based guided self-help CB intervention – ‘Get.Back’<br>• Weekly online modules<br>• 9 session & 1 booster session<br>• includeshomework assignments, interactive elements (emails, text messages, videos), reminders & exercises<br>• Guidance by a trained psychologist (e- Coach) with weekly feedback on the sessions<br><br>*Comparator: wait list*	• Information provision & patient education<br>• Training & rehearsal of psychological strategies<br>• Adherence support & lifestyle interventions<br>• eHealth-facilitated clinical review<br>• Remote monitoring with feedback & action plans	Body functions:<br>• Anxiety & depression - improved post treatment. This was maintained at 6-month follow-up for anxiety only<br>• Pain intensity & self-efficacy - unchanged<br><br>Activities & Participation:<br>• Disability - unchanged	
Telephone-based (n=15)							
Telephone-based	Thomas et al (2002)[110]<br><br>RCT	Knee OA<br><br>N = 786<br>(Exercise + telephone =121, exercise, telephone + placebo tablet = 114, exercise = 235, telephone = 160, placebo = 78, no intervention = 78)<br><br>Age = 62	Mixture of face-to-face & eHealth<br><br>Provider: research nurse<br><br>Frequency of in-person exercise: initially every 2 weeks for first 8 weeks, then at 6 month intervals<br>Frequency of telephone calls: monthly<br><br>Duration: 2 years<br><br>Follow-up: none	• Self-paced knee exercise program with graded elastic bands<br>• Telephone contact – monitor symptoms & advice on pain management<br>• Placebo tablet – dolomite health food product<br><br>*Comparator: standard care*	• Information provision & patient education<br>• Remote monitoring with feedback & action plans<br>• Adherence support & lifestyle interventions<br>• eHealth-facilitated clinical review<br>•	Body functions:<br>• Pain improved<br>• Anxiety & depression was not altered by any of the interventions<br><br>Activities & Participation:<br>• Knee specific physical function improved<br>• General physical function (SF-36) was not altered by any of the interventions	

Telephone-based	Thomas et al (2005)[111]<br><br>RCT	Knee OA<br><br>N = 786<br>(Exercise + telephone =121, exercise, telephone + placebo = 114, exercise = 235, telephone = 160, placebo = 78, no intervention = 78)<br><br>Age = 62	See Thomas et al[110]	• See Thomas et al[110]	• See Thomas et al[110]	Environmental factors:<br>• Cost of delivering the exercise program unlikely to be offset by any reduction in medical resource use	
Telephone-based	Damush et al (2003)[112]<br><br>RCT	Acute LBP<br><br>N = 211<br>(E = 106, C = 105)<br><br>Age = 45.5	Mixture of face-to-face & eHealth<br><br>Provider: Multiple Providers (Telephone calls: Research staff; In person classes: Session leaders)<br><br>Frequency: 3 in person classes – once per week Telephone calls to participants at 4, 6, & 8 weeks; thereafter once per month<br><br>Duration:<br>In person classes – 3 weeks<br>Telephone calls – 4 months<br><br>Follow-up: 12 months – See Damush et al [113]	Self-management program:<br>• 3 in-person classes – information about acute LBP, goal setting, problem solving & social support<br>• Class handouts – recommended exercises<br>• Classes on audiotape & a cassette player – provided if participant missed a class<br>• Physician letters of support – encouraging further participation in program<br>• Telephone follow-up – to discuss goal attainment, problem solve, set new goals<br><br>• *Comparator: standard care*	• Information provision & patient education<br>• Remote monitoring with feedback & action plans<br>• Adherence support & lifestyle interventions<br>• Training & rehearsal of psychological strategies<br>• eHealth-facilitated clinical review	Body Functions:<br>• Short term improvements in emotional functioning & self-efficacy	
Telephone-based	Damush et al (2003)[113]<br><br>RCT	Acute LBP<br><br>N = 211<br>(E = 106, C = 105)<br><br>Age = 45.5	See Damush et al[112] – 12 month follow-up	See Damush et al[112] – 12 month follow-up	• See Damush et al[112] – 12 month follow-up	Body Functions:<br>• Emotional functioning & self-efficacy improved & maintained for 1 year<br><br>Activities & Participation:	

						• Physical functioning improved & maintained for 1 year	
Telephone-based	Allen et al (2010)[114]<br><br>RCT	Hip or knee OA<br><br>N = 515 (self-management = 174, health education = 175, usual care = 174)<br><br>Age = 60.1	Mixture of face-to-face & eHealth<br><br>Provider: other (health counsellor)<br><br>Frequency: monthly<br><br>Duration: one year<br><br>Follow-up: none	Self-management Intervention<br>• Written & audio versions of osteoarthritis self-management educational material<br>• Exercise video<br>• Telephone call to discuss key points from educational material & answer any questions<br><br>*Comparator: health education intervention (attention control) & standard care*	• Information provision & patient education<br>• Remote monitoring with feedback & action plans<br>• eHealth-facilitated clinical review<br>•	Body Functions:<br>• Pain improved in self-management group<br>• Those in self-management group had greater improvement in self-efficacy than the health education group<br>• The self-management group did not differ from the other groups - Affect scores (mood & tension)<br><br>Activities & Participation:<br>• The self-management group did not differ from the other groups – global physical function<br>• Walking & bending improved<br><br>Environmental factors:<br>• Healthcare use did not differ across the groups	

Telephone-based	Iles et al (2011)[115]<br><br>RCT	Acute LBP<br><br>N = 30 (E = 15, C = 15)<br><br>Age = 39.5	Mixture of face-to-face & eHealth<br><br>Provider: Physiotherapist<br><br>Frequency of coaching: Once/week for first 4 weeks, with one further session 3 weeks later; frequency of physiotherapy sessions unspecified<br><br>Duration: Mixed (Coaching duration: Seven weeks, duration of physiotherapy unspecified)<br><br>Follow-up: week 12	Usual physiotherapy care + 5 telephone coaching sessions<br>• Coaching applied independently to physiotherapy, No correspondence between the treating therapist & coach<br><br>*Comparator:* usual physiotherapy care	• Information provision & patient education<br>• Remote monitoring with feedback & action plans<br>• eHealth-facilitated clinical review	Body Functions:<br>• Self-efficacy unchanged (no difference between groups)<br><br>Activities & Participation:<br>• Physical function did not differ between groups at 4 weeks, improved in E at week 12	
Telephone-based	Odole & Ojo (2013)[116]<br><br>Obs	Knee OA<br><br>N = 50 (E = 25, Clinic based group = 25)<br><br>Age = 55.5	Mixture of face-to-face & eHealth<br><br>Provider: Physiotherapist<br><br>Frequency: three times per week<br><br>Duration: six weeks<br><br>Follow-up: none	• Assessment & provision of copy of standardized HEP face-to-face in clinic<br>• Structured telephone call thrice-weekly to monitor & coach<br>• Exercise log book<br><br>*Comparator: clinic based physiotherapist administered standardized HEP*	• Information provision & patient education<br>• Adherence support & lifestyle interventions<br>• eHealth-facilitated clinical review<br>• Remote monitoring with feedback & action plans	Body functions:<br>• Pain improved (comparable to clinic-based group) post treatment<br><br>Activities & Participation:<br>• Physical functioning improved (comparable to clinic-based group) post treatment	
Telephone-based	Odole & Ojo (2014)[117]<br><br>Obs	Knee OA<br><br>N = 50 (E = 25, Clinic based group = 25)<br><br>Age = 55.5	See Odole & Ojo (2013)[116]	• See Odole & Ojo (2013)[116]	• See Odole & Ojo (2013)[116]	Activities & Participation:<br>• HRQOL – improved (comparable to clinic-based group) post treatment	
Telephone-based	Salisbury et al (2013)[36]<br><br>Mixed methods	Musculoskeletal conditions	Mixture of face-to-face & eHealth<br><br>Provider: Physiotherapist	'PhysioDirect' Telephone assessment and advice service	• Information provision & patient education	Quantitative:<br><br>Activities & Participation:	

		Quantitative:<br>N = 2256 (E = 1513, C = 743)<br><br>Age = 48<br><br>Qualitative:<br>N = 57<br><br>Age = 58	Frequency: Unstructured<br><br>Duration: Unspecified<br><br>Follow-up: none	• Initial assessment and advice<br>• Sent written advice about self-management and exercise<br>• Invited to phone back to discuss progress in 2-4 weeks<br>• If telephoned back, offered further advice +/- face-to-face appointment as indicated<br><br>*Comparator: usual care – face-to-face assessment and treatment*	• Adherence support & lifestyle interventions<br>• eHealth-facilitated clinical review<br>• Remote monitoring with feedback & action plans	• Physical component score was similar in both groups<br><br>Qualitative:<br>• Broadly acceptable<br>• Seen as first step in accessing treatment rather than replacing a face-to-face consultation.<br>• Many valued the faster access to advice & care & found the physiotherapists to be helpful during telephone consultations.<br>• However, some found the service to be impersonal & remote	
Telephone-based	Bennell et al (2017)[118]<br><br>RCT	Knee OA<br><br>N = 168 (E = 84, C = 84)<br><br>Age = 62.3	Mixture of face-to-face & eHealth<br><br>Provider: Multiple – musculoskeletal physiotherapists & 5 female telephone health coaches with health backgrounds (3 nurses, 1 occupational therapist, 1 health psychologist)<br><br>Frequency: Mixed - PT - weeks 1, 3, 7, 12 & 20<br>Coaching - weeks 2, 4, 8, 13, 21 & 25<br>± 6 additional calls can be made at any time during the 6 months<br><br>Duration: 6 months	5 x individual physiotherapy sessions & 6 to 12 telephone sessions with a health coach.<br><br>• Information booklet<br>• Exercise handouts<br>• Pedometer<br>• Exercise/physical activity log sheets<br><br>*Comparator: physiotherapy only (5 individual sessions)*	• Information provision & patient education<br>• Adherence support & lifestyle interventions<br>• Remote monitoring with feedback & action plans<br>• Training & rehearsal of psychological strategies<br>• eHealth-facilitated clinical review	Body functions:<br>• Pain improved in both E & C, no difference between groups – Addition of simultaneous coaching did not augment benefits of a PT prescribed PA program<br><br>Activities & Participation:<br>• Function improved in both E & C, no difference between groups – Addition of simultaneous	

			Follow-up: Month 12 & month 18			coaching did not augment benefits of a PT prescribed PA program	
Telephone-based	Hinman et al (2016)[38]<br><br>Qualitative	Knee OA<br><br>N = 6 patients with knee OA<br><br>Age = 62	See Bennell et al (2017)[118]	See Bennell et al (2017)[118]	See Bennell et al (2017)[118]	• Perceived as beneficial<br>• Balance needed with regards to structure & flexibility to promote individualization as indicated<br>• Video conferencing rather than telephone may facilitate relationship building	
Telephone-based	Schaller et al (2017)[119]<br><br>RCT	Chronic LBP<br><br>N = 412 (E = 201, C = 211)<br><br>Age = 51.9	Mixture of face to face & eHealth<br><br>Provider: multiple – movement coaches & inpatient rehabilitation (therapist specified)<br><br>Frequency: mixed<br>face to face contact – 3 times in 3 weeks/during inpatient stay<br>Web-based platform – unspecified<br>Inpatient rehabilitation –not specified<br>Telephone aftercare – twice in one month (week 8 & 12)<br><br>Duration:<br>Inpatient rehab – 3 weeks<br>3 face to face contacts – one hour<br>Web-based platform – 6 months	Multicomponenet intervention – 'Movement Coaching'<br><br>• Small group intervention – 23face to face sessions during inpatient rehabilitation<br>• Tailored telephone aftercare<br>• Web 2.0 intervention – did not meet criteria for internet based intervention – unstructured<br><br>*Comparator: low intensity control*<br>• 2 oral presentations (30 minutes) that were available for download after<br>During inpatient rehabilitation	• Information provision & patient education<br>• Remote monitoring with feedback & action plans<br>• Adherence support & lifestyle interventions<br>• eHealth-facilitated clinical review<br>•	Activities & Participation:<br>• Total or domain specific PA (leisure, workplace & transportation) unchanged<br>• Total PA worsened over 12 month study period<br><br>Environmental factors:<br>• Use of other healthcare services, medication use & sick leave change did not differ between groups at 6 month follow-up	

			Follow-up: 12 months				
Telephone-based	Goode et al (2018)[31]<br><br>RCT	Chronic LBP<br><br>N = 60 (PA + CB intervention = 20, PA = 20, C = 20)<br><br>Age = 70.3	Mixture of eHealth & face-to-face<br><br>Provider: multiple – physiotherapist & Exercise counsellor<br><br>Frequency: mixed. Physiotherapist – one call every 4 weeks – 3 in total. Exercise counsellor – week 1,2, 3, 5, 6, 7, 8, 10, 11, 12<br><br>Duration: 12 weeks<br><br>Follow-up: none	12 week telephone supported PA program & CB therapy intervention<br><br>PA program<br>• Telephone calls from physiotherapist<br>○ Addressing functional & clinical issues<br>• Telephone calls from exercise counsellor<br>○ PA goals<br>○ Barriers to goals<br>• Written instructions & pictures of exercise<br>• Exercise video – 'Taking control with exercise'<br><br>CB therapy intervention<br>• Received instruction in CB skills, with specific application to PA in each telephone-based session with the exercise counsellor<br>• Specific topics included – pacing, breathing relaxation, distraction, progressive muscle relaxation & cognitive restructuring<br><br>*Other comparator: wait list*	• Information provision & patient education<br>• Remote monitoring with feedback & action plans<br>• eHealth-facilitated clinical review<br>• Adherence support & lifestyle interventions<br>• Training & rehearsal of psychological strategies	Activities & Participation:<br>• Timed 'Up & Go' improved in both treatment groups compared to C – favored PA vs PA + CB intervention<br>• Activities of daily living self-report measure improved in both treatment groups compared to C – favored PA vs PA + CB intervention	
Telephone-based	Rutledge et al (2018)[120]<br><br>RCT	Chronic back pain<br><br>N = 66 (E = 33, C = 33)<br><br>Age = 53.3	Mixture of face-to-face & eHealth<br><br>Provider: other – mental health therapist<br><br>Frequency: 12 sessions over 8 weeks	Telephone based CB therapy<br>• First session was face to face – 2hours in duration<br>• Treatment 2 through 12 – via telephone, 30 mins in duration<br>• Manualized protocol	• Training & rehearsal of psychological strategies<br>• Information provision & patient education<br>• Remote monitoring with feedback & action plans<br>• Adherence support & lifestyle interventions	Body functions:<br>• Pain severity – improved<br><br>Activities & Participation:<br>• Disability – improved	

			Duration: 8 weeks<br><br>Follow-up: none	• Structured written materials that accompanied each phone session<br>• Relevant homework exercises were assigned at end of each session.<br><br>*Comparator: supportive care*<br>• *Education –‘Back Pain Help Book’*<br>• *Active listening by therapist*<br>*Supportive involvement – empathy, believing in the patient’s capacity to create own solutions & unconditional positive regard for patient*	• eHealth-facilitated clinical review		
Telephone-based	Swoboda (2019)[121]<br><br>Obs	Chronic LBP<br><br>N = 73<br><br>Age = 45.3	eHealth only<br><br>Provider: other – exercise physiologist<br><br>Frequency: monthly<br><br>Duration: 6 months<br><br>Follow-up: none	Monthly telephone calls to provide education & support for continued self-care<br>• Concurrent with usual care<br>Health coaches had no contact with medical team	• Information provision & patient education<br>• Adherence support & lifestyle interventions<br>• eHealth-facilitated clinical review	Body functions:<br>• Pain – improved<br><br>Activities & Participation:<br>• Disability – improved	
Telephone-based	Wang et al (2018)[122]<br><br>RCT	Knee pain<br><br>N = 390 (E = 206, C = 184)<br><br>Age = 39.5	Mixture of face to face & eHealth<br><br>Provider: other - facilitator<br><br>Frequency: mixed – once off group face to face meeting, monthly text messaging & one telephone based coaching session at week 12<br><br>Duration: 12 months	Low intensity self-management lifestyle intervention –the healthy lifestyle program for women - “HeLP-her’<br>• One face to fac group session<br>• Monthly text messages<br>• One telephone coaching session at week 12<br>• Program manual	• Information provision & patient education<br>• Adherence support & lifestyle interventions<br>• Remote monitoring with feedback & action plans<br>• eHealth-facilitated clinical review	Body functions:<br>• Lower risk of knee pain worsening in those that had knee pain in E compared to C at one year<br>• No effect on overall knee pain change for whole study sample	

			Follow-up: none	*Comparator: one general women’s health education session*				
Telephone-based	Williams et al (2018)[123]<br><br>RCT	Chronic LBP<br><br>N = 159 (E = 79, C = 80)<br><br>Age = 56.7	Mixture of face to face & eHealth<br><br>Provider: multiple – brief telephone advice – trained telephone interviewers, clinical consultation – physiotherapist, Coaching – delivered by a mixture of qualified health professional including dieticians, exercise physiologists & psychologists<br><br>Frequency:<br>mixed. One brief telephone advice session, one face to face clinical consultation, telephone coaching – 10 calls over 6 months<br><br>Duration:6 months<br><br>Follow-up: none	Healthy lifestyle intervention<br>• Brief telephone advice<br>• Face to face clinical consultation<br>• 6-month telephone based coaching service<br><br>*Comparator: standard care*	• Adherence support & lifestyle interventions<br>• Information provision & patient education<br>• Remote monitoring with feedback & action plans<br>• eHealth-facilitated clinical review	Body functions:<br>• Pain intensity - unchanged<br>• Depression & anxiety scores – unchanged over 6 months<br><br>Activities & Participation:<br>• Disability, stress & QOL – unchanged over 6 months	
Telephone-based	Williams et al (2019)[124]<br><br>RCT	Chronic LBP<br><br>N = 159 (E = 79, C = 80)<br><br>Age = 56.7	See Williams et al (2018)[123]	See Williams et al (2018)[123]	• See Williams et al (2018)[123]	Environmental factors:<br>• Healthcare costs, medication costs & absenteeism costs - improved	
Telephone-based	Gialanella et al (2019)[125]<br><br>RCT	Chronic neck pain<br><br>N = 100 (E = 50, C = 50)<br><br>Age = 57.1	Mixture of eHealth & face-to-face<br><br>Provider: multiple. Physiotherapist – outpatient rehabilitation, nurse-tutor - telephone based intervention<br><br>Frequency: mixed. Outpatient rehabilitation – 5 sessions per week for 2 weeks, telephone based intervention – one call every 2 weeks – 12 calls in total, ± unscheduled calls<br><br>Duration: 6.5 months	Telephone based intervention<br>• *Following 2 week exercise program in outpatient department.* Written & illustrated material explaining HEP<br>• Telephone based intervention following outpatient rehabilitation over following 6 months- 12 calls in total	• eHealth-facilitated clinical review<br>• Adherence support & lifestyle interventions<br>• Information provision & patient education<br>• Remote monitoring with feedback & action plans	Body functions:<br>• Pain – improved post treatment, not maintained at 12 months<br><br>Activities & Participation:<br>• Disability – improved post treatment, maintained at 12 months	

			Follow-up: 12 months	*Comparator: HEP without telephone based intervention following 2 week exercise program in outpatient department*<br>Written & illustrated material explaining HEP			
Telephone-based	Hinman et al (2019)[126]<br><br>RCT	Knee OA<br><br>N = 175 (E = 87, C = 88)<br><br>Age = 62.5	eHealth only<br><br>Provider: multiple. Existing service – nurse. Exercise advice & support – physiotherapist.<br><br>Frequency: mixed. Existing service – one call. Exercise advice & support – 5 – 10 calls over 6 months - Proposed schedule - weeks 2, 4, 8, 13 & 21<br><br>Duration: 6 months<br><br>Follow-up: 12 months	Physiotherapy led telephone based exercise advice & support intervention<br>• Exercise advice & support (5–10 consultations with a physiotherapist trained in behaviour change for a personalised strengthening & physical activity programme)<br>• Existing telephone service - ≥1 nurse consultation for self-management advice<br><br>*Comparator: existing service - ≥1 nurse consultation for self-management advice*	• Information provision & patient education<br>• Remote monitoring with feedback & action plans<br>• Adherence support & lifestyle interventions<br>• eHealth-facilitated clinical review	Body functions:<br>• Pain – did not improve at 6 months in E compared to C<br>• Improved pain self-efficacy at 6 & 12 months<br><br>Activities & Participation:<br>• Disability – improved at 6 months, not maintained at 12 months<br><br>Environmental factors:<br>• Intervention did not save health care resources	
Modality + telephone support (n=11)							
Internet based with telephone support	Buhrman et al (2004)[127]<br><br>RCT	Chronic lumbar, thoracic &/or cervical pain<br><br>N = 56 (E = 22, C = 29)<br><br>Age = 44.6	eHealth only<br><br>Provider: Graduate psychology students<br><br>Frequency of telephone calls: weekly<br>Frequency of internet based intervention: weekly<br><br>Duration: 8 weeks<br><br>Follow-up: 3 months	CB self-help treatment<br>• Internet-based CB program with psychological components + stretching & physical exercises<br>• Weekly telephone support<br>• Pain diaries<br>• Coping diaries<br>• CD for applied relaxation<br><br>*Comparator: Wait list control*	• eHealth-facilitated clinical review<br>• Information provision & patient education<br>• Training & rehearsal of psychological strategies<br>• Adherence support & lifestyle interventions<br>• Remote monitoring with feedback & action plans	Body functions:<br>• Catastrophizing & coping strategies improved<br>• Some improvement was found in both E & C<br>• Follow-up results indicated that some improvement was maintained<br>• Anxiety & depression – no	

						significant main effects or interaction for group & time were found	
Internet based with telephone support	Buhrman et al (2011)[64]<br><br>RCT	Chronic back pain<br><br>N = 54<br><br>Age = 43.2	Mixture of face-to-face & eHealth<br><br>Provider: Clinical psychologists<br><br>Frequency of internet based intervention: Weekly; Frequency of telephone support: Once after two weeks of intervention<br><br>Duration: Twelve weeks<br><br>Follow-up: none	Internet-based CBT self-help management program<br>• 8 modules<br>• Submission of weekly reports (e.g., homework assignments)<br>• Therapist feedback/encourageme nt via email weekly<br><br>*Comparator: waiting list*	• Information provision & patient education<br>• Remote monitoring with feedback & action plans<br>• eHealth-facilitated clinical review<br>• Training & rehearsal of psychological strategies<br>• Adherence support & lifestyle interventions	Body Functions:<br>• Catastrophizing improved<br>• However, most outcomes did not indicate a positive treatment outcome<br><br>Activities & Participation:<br>• QOL improved	
Internet based with telephone support	Buhrman et al (2013)[65]<br><br>RCT	Chronic musculoskeletal conditions<br><br>N = 76 (E = 38, C = 38)<br><br>Age = 49.1	eHealth only<br><br>Provider: Graduate psychology students<br><br>Frequency of internet-based program: Weekly<br>Frequency of telephone calls: Following completion of section 3 & at seven weeks<br><br>Duration: seven weeks<br><br>Follow-up: six months	Guided internet-based ACT intervention<br><br>• Seven sections consisting of information, relevant metaphors & mindfulness exercises<br>• Weekly homework assignment via secure platform<br>• Weekly therapist feedback via secure platform<br>• Two structured telephone calls to motivate & encourage<br><br>*Comparator: moderated online discussion group*	• Information provision & patient education<br>• Training & rehearsal of psychological strategies<br>• Adherence support & lifestyle interventions<br>• Remote monitoring with feedback & action plans<br>• eHealth-facilitated clinical review	Body functions:<br>• Pain-related distress, anxiety & depression improved<br>• Maintenance of improvement exhibited at 6-month follow-up<br><br>Activities & Participation:<br>• QOL unchanged	

Internet based with telephone support	Bendelin et al (2020)[128]<br><br>Qualitative	Chronic musculoskeletal conditions<br><br>N = 11<br><br>Age = 46	See Buhrman et al (2013)[65]	See Buhrman et al (2013)[65]	See Buhrman et al (2013)[65]	• Adjusting treatment format based on the different needs of chronic pain patients may improve engagement & change<br>• Directing therapist guidance to the process of goal setting & exploring pain-related thoughts & feelings may be helpful	
Internet based with telephone support	Dear et al (2013)[69]<br><br>RCT	Chronic musculoskeletal conditions<br><br>N = 62 (E= 31, C = 31)<br><br>Age = 49	eHealth only<br><br>Provider: Clinical psychologist<br><br>Frequency of internet-based programme: approx. weekly (every 7 – 10 days)<br>Frequency of telephone call or email: weekly<br><br>Duration: Eight weeks<br><br>Follow-up: Three months	Clinician-guided internet-based CBT program – ‘The Pain Course’<br><br>• Five modules<br>• Homework tasks<br>• Additional resources<br>• Weekly e-mail or telephone contact from a Clinical Psychologist,<br>• Automated e-mails<br><br>*Comparator: waiting list*	• Information provision & patient education<br>• Training & rehearsal of psychological strategies<br>• Remote monitoring with feedback & action plans<br>• eHealth-facilitated clinical review<br>• Adherence support & lifestyle interventions	Body functions:<br>• Improved pain, anxiety & depression post treatment, maintained at 3-month follow-up<br><br>Activities & Participation:<br>• Improved disability post treatment, maintained at 3-month follow-up	
Internet based with telephone support	Dear et al (2015)[68]<br><br>RCT	Chronic musculoskeletal conditions<br><br>N = 490 (Regular contact = 143, Optional contact = 141, no contact = 131, waitlist control = 75)<br><br>Age = 50	See Dear et al (2013)[69]	See Dear et al (2013)[69] for intervention – regular contact<br><br>Optional Contact:<br>• Informed that clinician was available & could be contacted on an ‘as needed’ basis<br>• Clinician did not initiate contact<br><br>No Contact:	• See Dear et al (2013)[69]	Body functions:<br>• Pain, anxiety & depression improved in all 3 treatment groups & this was sustained or further improved at 3-month follow-up.<br><br>Activities & Participation:	

				• No contact unless technical issues or mental health emergency<br><br>*Other comparator: waitlist control*		• Disability improved in all 3 treatment groups & further improved at 3-month follow-up.<br><br>Environmental factors:<br>• Improvements in medication & healthcare use across treatment & control groups	
Internet based with telephone support	Friesen et al (2017) [58]<br><br>RCT	Fibromyalgia<br><br>N = 60 (E = 30, C = 30)<br><br>Age = 47.5	See Dear et al (2013)[69]<br><br>Follow-up: 4 weeks	See Dear et al (2013)[69]<br><br>*Comparator: waitlist control*	See Dear et al (2013)[69]<br>•	Body functions:<br>• Improved symptom severity, depression, pain & fear of pain<br>• Smaller effects observed for anxiety<br>• Changes maintained at 4-week follow-up<br><br>Activities & Participation:<br>• Small improvement in physical health post treatment, maintained at 4 weeks	
Internet based with telephone support	Dear et al (2018)[67]<br><br>RCT	Chronic musculoskeletal conditions<br><br>See Dear et al (2015)[68]<br><br>12 & 24 month follow-up	See Dear et al (2015)[68]	See Dear et al (2015)[68]	See Dear et al (2015)[68]	Body functions:<br>• Improvements in pain, anxiety & depression were maintained at 12 & 24 months.<br>• No differences between treatment groups were noted<br><br>Activities & Participation:	

						• Improvements in disability were maintained at 12 & 24 months for disability.<br>• No differences between treatment groups were noted	
Internet based with telephone support	Dear et al (2018)[66]<br><br>Obs	Chronic musculoskeletal conditions<br><br>N = 43<br><br>Age = 58	See Dear et al (2013)[69]	See Dear et al (2013)[69]	See Dear et al (2013)[69]	Body functions:<br>• Depression – improved<br>• Anxiety levels – unchanged<br>• Self-efficacy – improved between pre- & post-treatment but this reversed between post treatment & 3-month follow-up<br><br>Activities & Participation:<br>• Disability – improved between pre-treatment & 3 month follow-up but not between pre- & post-treatment	
Internet based with telephone support	Buhrman et al (2015)[74]<br><br>RCT	Chronic musculoskeletal conditions with comorbid anxiety & depression<br><br>N = 52 (E = 28, C = 24)<br><br>Age: 50.7	eHealth only<br><br>Provider: Graduate psychology students<br><br>Frequency of internet-based: weekly<br>Frequency of telephone support: at 4 weeks +/- 8 weeks if participant agreed<br><br>Duration: 8 weeks<br><br>Follow-up: 1 year	Guided internet-based CBT intervention<br>• 8 sections & assignments, individualized according to screening interview information & baseline questionnaires<br>• Therapist feedback via secure platform<br>• Telephone support on 1-2 occasions<br>• Text message reminders if	• Information provision & patient education<br>• Remote monitoring with feedback & action plans<br>• eHealth-facilitated clinical review<br>• Training & rehearsal of psychological strategies<br>• Adherence support & lifestyle interventions	Body functions:<br>• Pain catastrophizing improved<br>• Anxiety & depression improved<br>• One-year follow-up showed maintenance of improvements<br><br>Activities & Participation:	

				assignments incomplete<br><br>*Comparator: moderated online discussion forum*		• Disability improved, maintained at 1 year follow-up	
Internet based with telephone support	Peters et al (2017) [81]<br><br>RCT	Chronic musculoskeletal conditions<br><br>N = 284 (internet positive psychology intervention = 117, internet CB intervention = 116,, C = 51)<br><br>Age= 48.9	eHealth only<br><br>Provider: Graduate psychology students<br><br>Frequency: Mixed internet based intervention – weekly. Telephone support - weeks 1, 3, 5 & 7)<br><br>Duration: 8 weeks<br><br>Follow-up: 6 months (excludes C)	Internet based positive psychology self-help program – ‘Happy Despite Pain’<br>• 8 modules<br>• Self-compassion diary & letter to oneself<br>• Gratitude exercises<br>• Savoring diary<br>• Best possible self exercise<br>• Telephone support<br>• Email support (weeks 2, 4, 6, & 8)<br><br>Internet based CB intervention<br>• Based on Buhrman et al (2004)[127]<br>• 8 modules<br>• Telephone support<br>• Email support (weeks 2, 4, 6, & 8)<br><br>*Other comparator: waitlist control*	• Training & rehearsal of psychological strategies<br>• Information provision & patient education<br>• eHealth-facilitated clinical review<br>•	Body functions:<br>• Depression improved in both treatment groups compared to C post treatment.<br>• No differences between both treatment groups at 6 months<br>• Anxiety & catastrophizing improved for both groups compared to C post treatment, maintained at 6-month follow-up<br>• Pain intensity – unchanged<br><br>Activities & Participation:<br>• Improvements in physical impairments in both treatment groups, similar to improvements in C	
Internet based with telephone support	Geraghty et al (2018)[129]<br><br>RCT	LBP<br><br>N = 87 (internet intervention + PT support = 29, internet intervention + usual care = 30, C = 28)<br><br>Age = 58	eHealth only<br><br>Provider: Physiotherapist<br><br>Frequency: mixed. Internet based intervention – weekly. Telephone support – 3 calls over a 4-week period	Internet based intervention – ‘SupportBack’<br>• 6 sessions<br>• Graded goal setting<br>• Self-monitoring<br>• Tailored feedback<br>• Automated weekly email reminders<br>• Telephone support	• Information provision & patient education<br>• Remote monitoring with feedback & action plans<br>• Adherence support & lifestyle interventions<br>• Training & rehearsal of psychological strategies	Body functions:<br>• Pain intensity improved in all 3 groups<br>• Greatest improvement in SupportBack + PT support vs	

			Duration: 6 weeks<br><br>Follow-up: 3 months	SupportBack plus usual care<br><br>*Other comparator: standard care*	• eHealth-facilitated clinical review	SupportBack + usual care<br>• Pain catastrophizing worsened in both C & SupportBack + PT support<br>• Fear avoidance beliefs improved in all 3 groups<br><br>Activities & Participation:<br>• Disability improved in all 3 groups<br>• Greater improvement in disability in SupportBack + PT support vs. C, compared to SupportBack + usual care vs. C.	
Internet based with telephone support	Geraghty et al (2019)[130]<br><br>Qualitative	LBP<br><br>N = 15<br><br>Age = 56.2	See Geraghty et al (2018)[129]	See Geraghty et al (2018)[129]	See Geraghty et al (2018)[129]	• Feasible<br>• Highlighted importance of self-management & PA while providing reassurance<br>• Prompts & reminders helped with the behavioral change & maintenance<br>• Pre-existing condition or severity acted as barriers<br>• Less useful for those with mild LBP<br>• Telephone support provided additional	

						reassurance & motivation	
Internet based with telephone support	Higgins et al (2020)[34]<br><br>Mixed methods	Chronic LBP<br><br>N = 15 (Qualitative)<br><br>Age = 50.9<br><br>N = 58 (Quantitative)<br><br>Age = 55	eHealth only<br><br>Provider: other (study staff)<br><br>Frequency: weekly<br><br>Duration: 10 weeks<br><br>Follow-up: none	Internet-based self-management program – 'PAIN Ease' – Pain eHealth for Activity, Skills and Education<br>• Screening via telephone or in-person<br>• Self-directed – no clinician involvement<br>• Weekly telephone calls by study staff<br>• Step count<br>• Sleep tracking<br>• Relaxation practice<br>• Coping skills modules<br>• 'Test Your Knowledge' quizzes	• Information provision & patient education<br>• Remote monitoring with feedback & action plans<br>• Adherence support & lifestyle interventions<br>• Training & rehearsal of psychological strategies	Quantitative:<br><br>Body functions:<br>• Within-subject improvement for pain interference, mood and depression<br><br>Qualitative:<br>• May benefit from technology-delivered interventions, which may also reduce pain interference<br>• Internet based intervention is feasible & satisfactory	
Mobile phone application with telephone-support	Amorim et al (2019)[131]<br><br>RCT	Chronic LBP<br><br>N = 68 (E = 34, C = 34)<br><br>Age = 58.3	Mixture of face to face & eHealth<br><br>Provider: Health coaches<br><br>Frequency: mixed. Face to face assessment & interview occurred once at beginning of intervention. Coaching – every 2 weeks, mobile phone application – unstructured.<br><br>Duration: 6 months<br><br>Follow-up: none	Patient centred PA intervention<br>• Information booklet<br>• Individually tailored PA plan with guidance from coach<br>• Face to face assessment & interview<br>• Telephone coaching<br>• Activity tracker<br>• Mobile phone application – IMPACT app<br>• Weekly personalized messages<br><br>*Comparator: standard care*	• eHealth-facilitated clinical review<br>• Remote monitoring with feedback & action plans<br>• Adherence support & Lifestyle interventions<br>• Information provision & patient education<br>• Training & rehearsal of psychological strategies	Body functions:<br>• Pain - unchanged<br><br>Activities & Participation:<br>• Disability - unchanged<br><br>Environmental factors:<br>• 38% reduced rate of care seeking in E (p = 0.14)	

Interactive voice response with telephone-support	Kroenke et al (2014)[73]<br><br>RCT	Chronic musculoskeletal conditions<br><br>N = 250 (E = 124, C = 126)<br><br>Age = 55.2	Mixture of eHealth & face-to-face<br><br>Provider: multiple (nurse care manager & physician pain specialist with primary care physician<br><br>Frequency of IVR: weekly for the first month, every other week for months 2 & 3, & monthly for months 4 through 12<br>Frequency of telephone support: Scheduled for 1 month & 3 months. Could also be arranged<br><br>Duration: 12 months<br><br>Follow-up: none	Telephone-delivered collaborative care management intervention - ‘Stepped Care to Optimize Pain Care Effectiveness (SCOPE)’<br>• IVR automated symptom monitoring<br>• Optimised analgesic management consisting of 1 face-to-face meeting with nurse & 2 scheduled telephone support calls<br><br>*Comparator: standard care*	• eHealth-facilitated clinical review<br>• Adherence support & lifestyle interventions<br>• Remote monitoring with feedback & action plans	Body functions:<br>• Pain improved<br>• Depression improved<br>• Anxiety & sleep unchanged<br><br>Activities & Participation:<br>• Physical functioning unchanged<br><br>Environmental factors:<br>• Opioid use unchanged	
Interactive voice response with telephone-support	Guilkey et al (2016)[33]<br><br>Mixed methods	Chronic musculoskeletal conditions<br><br>N = 124 (Telecare use - ASM reports & nurse calls)<br><br>Age = 55<br><br>N = 108 (Satisfaction end-of-study survey)<br><br>N = 118 (End-of-study interview)	See Kroenke et al (2014)[73]	See Kroenke et al (2014)[73]	See Kroenke et al (2014)[73]	• High patient satisfaction with & adherence to both the automated & the nurse component of the telecare intervention<br>• Only a minority of the ASM reports had patient responses that required an unscheduled nurse contact; may increase efficiency, allowing provider to focus more on patients who are responding or having side-effects for example	
Interactive voice	Kroenke et al (2019)[72]	Chronic musculoskeletal conditions	Mixture of face-to-face & eHealth	Comprehensive symptom management	• Remote monitoring with feedback & action plans	Body functions:	

response with telephone-support & internet-based	RCT	N = 294 (E = 147, C = 147)<br><br>Age = 57.4	Provider: multiple. Nurse-physician team<br>+/- psychologist / psychiatrist<br><br>Frequency: mixed. Scheduled calls – baseline, 1, 4 & 12 weeks<br>Nurse & physician met once weekly<br>The monitoring schedule was weekly for the first month, twice a month for months 2–6, & monthly for months 7–12.<br><br>Duration: 12 months<br><br>Follow-up: none	• Automated monitoring via IVR<br>• 9 internet based self-management modules<br>• Collaborative care management by a nurse-physician team – physician & nurse met weekly; nurse linked with patient via telephone – stepped care analgesic algorithm in line with Kroenke et al (2014)[73]<br><br>*Comparator – Automated symptom management*<br>• *Automated monitoring*<br>• *9 internet based self-management modules*	• eHealth-facilitated clinical review<br>• Training & rehearsal of psychological strategies<br>• Information provision & patient education<br>• Adherence support & lifestyle interventions	• Both interventions (relying heavily on telecare but differing in resource intensity) – improved pain & mood<br>• However, E (Comprehensive symptom management) was superior<br><br>Activities & Participation:<br>• Social functioning & disability – improved in E<br><br>Environmental factors:<br>• Opioid use improved in both groups, similar between groups<br>• Health services use was similar in both groups	
Mobile Phone Application (n=8)							
Mobile Phone Application	Lee et al (2017) [132]<br><br>RCT	Chronic neck pain<br><br>N = 20 (E = 11, C = 9)<br><br>Age = 27.3	Mixture of face-to-face & eHealth<br><br>Provider: Physiotherapist<br><br>Frequency: twice per week<br><br>Duration: 8 weeks<br><br>Follow-up: none	Mobile phone application based neck exercise in the workplace environment<br><br>• McKenzie neck exercise program<br>• Weekly text messages about neck pain management<br><br>*Comparator:*	• Information provision & patient education<br>• Remote monitoring with feedback & action plans<br>• Adherence support & lifestyle interventions	Body functions:<br>• Pain intensity improved<br>• Fear avoidance beliefs partially improved<br><br>Activities & Participation:<br>• Function improved<br>• Muscle strength & QOL partially improved	

				• *One education session on neck pain management*<br>• *Brochure indicating correct posture,*<br>*Weekly text messages about neck pain management*			
Mobile Phone Application	Chhabra et al (2018)[41]<br><br>RCT	Chronic LBP<br><br>N = 93 (E = 45, C = 48)<br><br>Age = 41.2	Mixture of face-to-face & eHealth<br><br>Provider: other - physician<br><br>Frequency: daily<br><br>Duration: 12 weeks<br><br>Follow-up: none	Mobile phone application – ‘Snapcare’<br>• Individualized daily PA goals<br>• Emphasis on improving function<br>• Auto-generated app notifications & reminders<br>• Gamification – system of rewards<br>• Written prescription from physician<br><br>*Comparator: standard care (includes written prescription from physician)*	• Remote monitoring with feedback & action plans<br>• Adherence support & lifestyle interventions<br>•	Body functions:<br>• Both groups showed improvement in pain, with no between group difference<br><br>Activities & Participation:<br>• Disability improved in E compared to C	
Mobile Phone Application	Lo et al (2018)[55]<br><br>Obs	Chronic widespread musculoskeletal conditions<br><br>(Chronic neck & back pain)<br><br>N = 158<br><br>Age = Not reported	eHealth only<br><br>Provider: multiple – physiotherapists, rehabilitation doctor, clinical scientist.<br><br>Frequency: unstructured<br><br>Duration: unspecified<br><br>Follow-up: none	Artificial intelligence mobile phone application – “Well Health Mobile App’<br>• Tailored exercise rehabilitation program based on symptoms-self report questionnaire<br>• Artificial intelligence algorithm determined HEP<br>• Re-assessment every 2 weeks<br>• Exercise videos – points rewards when full length of video played<br>• All users could contact medical team	• eHealth-facilitated clinical review<br>• Information provision & patient education<br>• Remote monitoring with feedback & action plans<br>• Adherence support & lifestyle interventions<br>•	Body functions:<br>• Pain intensity - improved	

				• Points-based reward utilized to promote engagement<br>Educational material via social media platform			
Mobile Phone Application	Mecklenburg et al (2018)[133]<br><br>RCT	Chronic knee pain<br><br>N = 162 (E = 101, C = 61)<br><br>Age = 46.5	eHealth only<br><br>Provider: N/A - eHealth modality fully automated<br><br>Frequency: mixed. Exercise therapy – 3-4 times per week, education – once per week, symptom logging – twice per week, activity tracking – at least 3 times per week, CBT – frequency not defined, team & coach interaction – at least once per week<br><br>Duration: 12 weeks<br><br>Follow-up: none	Mobile phone based digital care program – ‘Hinge Health Digital Care Program’<br>• Sensor guided exercise therapy<br>• Education<br>• CB therapy<br>• Weight loss<br>• Psychosocial support via personal coach & team-based interaction<br>• Activity tracking<br><br>*Comparator: 3 education pieces regarding self-care for chronic knee pain*	• Information provision & patient education<br>• Remote monitoring with feedback & action plans<br>• Adherence support & lifestyle interventions<br>• Training & rehearsal of psychological strategies<br>• eHealth-facilitated clinical review	Body functions:<br>• Pain & stiffness improved compared to C post treatment<br><br>Activities & Participation:<br>• Function improved compared to C post treatment<br><br>Environmental factors:<br>• Likelihood of having surgery in the next 1 year, 2 years & 5 years compared to C – reduced/ improved	
Mobile Phone Application	Shebib et al (2019)[134]<br><br>RCT	LBP<br><br>N = 177 (E = 113, C = 64)<br><br>Age = 43	See Mecklenburg et al (2018)[133]	See Mecklenburg et al (2018)[133]	• See Mecklenburg et al (2018)[133]	Body functions:<br>• Pain – improved<br><br>Activities & Participation:<br>• Disability – improved	

Mobile Phone Application	Toelle et al (2019)[135]<br><br>RCT	Back pain<br><br>N = 101 (E = 53, C = 48)<br><br>Age = 42	Mixture of eHealth & face-to-face<br><br>Provider: multiple – physical examination - medical doctor, within chat function of app – physiotherapist/ sports scientist<br><br>Frequency: 4 times per week<br><br>Duration: 3 months<br><br>Follow-up: none	Mobile phone application based MDT back pain treatment – ‘Kaia App’<br>• Back pain specific education<br>• Physiotherapy/ physical exercise<br>• Mindfulness & relaxation<br>• Pain diary<br><br>*Comparator: 6 individual physiotherapy sessions over 6 weeks plus high-quality online education*	• Information provision & patient education<br>• Training & rehearsal of psychological strategies<br>• eHealth-facilitated clinical review<br>• Adherence support & lifestyle interventions	Body functions:<br>• Pain intensity – improved<br><br>Activities & Participation:<br>• Functional ability & well-being – unchanged<br><br>Environmental factors:<br>• No group different regarding pain medication	
Mobile Phone Application	Huber et al (2017)[136]<br><br>Obs - retrospective	LBP<br><br>N = 180<br><br>Age = 33.9	See Toelle et al (2019)[135]	See Toelle et al (2019)[135]	• See Toelle et al (2019)[135]	Body functions:<br>• Pain – improved	
Mobile Phone Application	Yang et al (2019)[137]<br><br>RCT	Chronic LBP<br><br>N = 8 (E = 5, C = 3)<br><br>Age = 42.7	Mixture of face-to-face & eHealth<br><br>Provider: physiotherapist<br><br>Frequency: mixed. HEP 4 times daily. Frequency of physiotherapy not speciified<br><br>Duration: 4 week<br><br>Follow-up: none	Mobile phone based self-management plus physiotherapy – app called ‘Pain Care’<br>• Exercise reminder via app<br>• Pain diary via app<br>• Sharing inputted data via email<br>• Can tailor reminders via App<br><br>*Comparator: physiotherapy only*	• Remote monitoring with feedback & action plans<br>• eHealth-facilitated clinical review<br>• Adherence support & lifestyle interventions	Body functions:<br>• Self-efficacy improved<br>• Pain – positive trend but no within- or between-group differences<br><br>Activities & Participation:<br>• Bodily pain & mental health aspects of health related QOL - improved	

						• Disability – improved	
Mobile Phone Application	Hasenöhrl et al (2020)[37]<br><br>Mixed methods	LBP<br><br>N = 27 (Quantitative analysis of secondary data)<br><br>Age = 44.8<br><br>N = 16 (Qualitative semi structured interviews)<br><br>Age = 45.1	Mixture of face-to-face & eHealth<br><br>Provider: other (orthopaedic specialist)<br><br>Frequency: unstructured<br><br>Duration: 4 weeks<br><br>Follow-up: none	Mobile phone application<br>• Videos of 48 therapeutic exercises<br>• App decided exercises depending on acute or chronic diagnosis inputted by physician<br>• Physician could modify suggested exercises<br>• After finishing each exercise, patients could give feedback via traffic light system – if 'red' or excessive amount of 'yellow', doctor was alerted	• eHealth-facilitated clinical review<br>• Adherence support & lifestyle interventions	Quantitative:<br><br>Activities & Participation:<br>• Disability and QOL domains physical functioning, bodily pain and vitality improved<br><br>Qualitative:<br>• Feasible & well-accepted<br>• Pilot data points towards efficacy of the intervention.	
Mobile Phone Application	Hauser-Ulrich et al (2020)[60]<br><br>RCT	Chronic musculoskeletal conditions<br><br>N = 102 (E = 59, C = 43)<br><br>Age = 44	eHealth only<br><br>Provider: N/A - eHealth modality fully automated<br><br>Frequency: daily<br><br>Duration: 8 weeks<br><br>Follow-up: none	Automated mobile phone application-based CB therapy chatbot - painSELfMAnagement (SELMA)<br>• Pain diary<br>• Delivers psychoeducation from day 1 to 21 in daily text messages<br>• Day 22 until the end, SELMA delivers CB therapy intervention modules<br><br>*Comparator: wait list*	• Training & rehearsal of psychological strategies<br>• Information provision & patient education<br>• Adherence support & lifestyle interventions	Body functions:<br>• Pain intensity-improved<br><br>Activities & Participation:<br>• Well-being improved<br>• Pain related impairment – small improvement, similar to C	
Interactive voice response (n=3)							
Interactive voice response	Naylor et al (2002)[59]<br><br>Obs	Chronic musculoskeletal conditions<br><br>N = 11	Mixture of face-to-face & eHealth<br><br>Provider: Other (therapist)	10 weeks of group CB therapy followed by 4 months of IVR<br><br>CB therapy group	• Information provision & patient education<br>• Remote monitoring with feedback & action plans	Body functions:<br>• Pain, mental health composite score &	

		Age = 46.7	Frequency: CBT group: weekly<br>IVR: daily self-monitoring, monthly feedback<br><br>Duration CBT group: 10 weeks<br>Duration IVR: 4 months<br><br>Follow-up:	• Relaxation Techniques & Cognitive Coping Strategies<br>• Challenging pain beliefs<br>• Pain diaries<br>• Spouses/significant others invited to attend week 8<br><br>IVR<br>• 21 item daily questionnaire – self monitoring<br>• Didactic skills review<br>• Guided behavioral rehearsal of pain coping skills<br>Monthly therapist feedback	• Adherence support & lifestyle interventions<br>• Training & rehearsal of psychological strategies	catastrophizing improved<br><br>Activities & Participation:<br>• Disability improved	
Interactive voice response	Naylor et al (2008)[61]<br><br>RCT	Chronic musculoskeletal conditions<br><br>N = 55 (E = 29, C = 26)<br><br>Age = 46.5	Mixture of face-to-face & eHealth<br><br>Provider: - other (therapist)<br><br>Frequency of group CBT: weekly, Frequency of IVR: daily self-monitoring with monthly therapist feedback<br><br>Duration of group CBT: 11 weeks<br>Duration of IVR: 4 months<br><br>Follow-up: 8 months post CBT group, 4 months post IVR	Maintenance enhancement following group CBT<br>• Daily self-monitoring questionnaire<br>• Didactic review of coping skills<br>• Pre-recorded behavioral rehearsals of coping skills<br>• Monthly personalized feedback messages<br><br>*Comparator: standard care*	• Remote monitoring with feedback & action plans<br>• Training & rehearsal of psychological strategies<br>• Information provision &<br>• Adherence support & lifestyle interventions<br>• eHealth-facilitated clinical review	Body Functions:<br>• Improved pain & pain coping<br><br>Activities & Participation:<br>• Physical activity & performance (SF36 Physical Composite Score) improved	
Interactive voice response	Naylor et al (2010)[62]<br><br>RCT	Chronic musculoskeletal conditions<br><br>N = 55 (E = 29, C = 26)<br><br>Age = 46.5	See Naylor et al (2008)[61]	See Naylor et al (2008)[61]	• See Naylor et al (2008)[61]	Environmental factors:<br>• Opioid & NSAID medication use improved	
Interactive voice response	Heapy et al (2017)[138]<br><br>Noninferiority randomized trail	Chronic LBP<br><br>N = 125 (E = 62, in-person CB therapy= 63)	Mixture of face-to-face & eHealth<br><br>Provider: Multiple – clinical psychologists or advanced	IVR-CBT<br>• Self-help manual<br>• Coping skill practice & meaningful activity goals	• Training & rehearsal of psychological strategies<br>• Remote monitoring with feedback & action plans	Body functions:<br>• Pain intensity improved in both groups at month	

		Age = 58	practice psychiatric nurse practitioners<br><br>Frequency: Mixed – weekly IVR-CB therapy/in-person CB therapy; daily IVR assessment<br><br>Duration: 11 weeks<br><br>Follow-up: month 3, 6 & 9	• Pedometer-assisted graduated walking program<br>• IVR assessment – pain intensity, step count & sleep duration<br>• 10 weekly prerecorded therapist feedback based on their IVR-reported activity via IVR<br><br>Comparator: in-person CB therapy<br>• Self-help manual – shortened version<br>• Coping skill practice meaningful activity goals<br>• Pedometer-assisted graduated walking program<br>• IVR assessment - pain intensity, step count & sleep duration<br>10 individual weekly in-person CB therapy	• eHealth-facilitated clinical review<br>• Information provision & patient education<br>• Adherence support & lifestyle interventions	3 & 6 but not at month 9<br>• IVR-CBT was noninferior to in-person CBT for pain intensity improvement<br>• Sleep quality – improved in both groups at 3 months<br><br>Activities & Participation:<br>• Physical functioning & physical QOL – improved in both groups, no differences between groups	
Video tele conferencing (n=2)							
Video tele conferencing	Wong et al (2005) [32]<br><br>Obs	Knee pain<br><br>N = 22<br><br>Age = 75	Mixture of face-to-face & eHealth<br><br>Provider: Physiotherapist<br><br>Frequency: weekly<br><br>Duration: 12 weeks<br><br>Follow-up:	Weekly centre-based exercise programme via videoconferencing<br>• Reinforcement of HEP – three times per week<br>• Self-management education<br>• Peer support<br>• Exercise logbook	• Information provision & patient education<br>• eHealth-facilitated clinical review<br>• Adherence support & lifestyle interventions	Body Functions:<br>• Pain & balance improved<br><br>Activities & Participation:<br>• Physical function, Quad strength, QOL & functional performance (TUAG) improved	

Video tele conferencing	Herbert et al (2017)[51]<br><br>Noninferiority randomized trail	Chronic musculoskeletal conditions<br><br>N = 129 (in-person = 65, video teleconferencing = 64)<br><br>Age = 52	Mixture of face-to-face & eHealth<br><br>Provider: Graduate psychology students<br><br>Frequency: weekly<br><br>Duration: 8 weeks<br><br>Follow-up: 6 months	Video-teleconferencing ACT intervention<br>• Face-to-face medical evaluation initially<br>• 8 60-minute weekly sessions<br>• Emphasis on at-home assignments<br><br>In-person ACT intervention<br>• Face-to-face medical evaluation initially<br>• 8 60-minute weekly sessions<br>• Emphasis on at-home assignments	• Training & rehearsal of psychological strategies<br>• eHealth-facilitated clinical review<br>• Information provision & patient education<br>• Remote monitoring with feedback & action plans<br>• Adherence support & lifestyle interventions<br>•	Body functions:<br>• Pain interference, pain severity, depression & pain-related anxiety - improved<br>• Sleep quality - unchanged<br><br>Activities & Participation:<br>• Physical QOL – improved, no significant between group differences<br>• Activity levels – greater improvement in in-person group from baseline to 6 month follow-up	
VR (n=1)					•		
VR	Darnall et al (2020)[139]<br><br>RCT	Chronic LBP or Fibromyalgia<br><br>N = 74 (E = 39, C = 35)<br><br>Age = Not available	eHealth only<br><br>Provider: N/A - eHealth modality fully automated<br><br>Frequency: daily<br><br>Duration: 3 weeks<br><br>Follow-up: none	Skills based self-administered VR program<br>• Both groups had same didactic content<br><br>Three main content categories:<br>• Skills rooted in pain CBT<br>• Relaxation training<br>○ Optimised in VR group with visual bio-feedback<br>• Mindfulness<br><br>*Comparator: audio-only version of the VR program*	• Information provision & patient education<br>• Training & rehearsal of psychological strategies<br>• Adherence support & lifestyle interventions	Body functions:<br>• Average pain intensity, sleep & mood improved<br>• Pain self-efficacy & catastrophising improved similarly in both E & C<br><br>Activities & Participation:<br>• Pain-related interference with activity & stress improved	

Internet-based with video conferencing (n=1)							
Internet-based with video conferencing	Bennell el al (2017)[140]<br><br>RCT	Chronic knee pain<br><br>N = 148 (E = 74, C = 74)<br><br>Age = 61.2	Via eHealth only<br><br>Provider: physiotherapists<br><br>Frequency: Mixed – PCST – weekly<br>Physiotherapy via skype – approx. fortnightly (7 over 12 weeks)<br><br>Duration: 3 months<br><br>Follow-up: 6 months	Exercise + PCST<br>• Online educational material<br>• 7 individual physiotherapy sessions vis skype<br>• HEP<br>• PCST – 8 weekly sessions plus monthly reminder emails<br><br>*Comparator: online educational material*	• Information provision & patient education<br>• eHealth-facilitated clinical review<br>• Training & rehearsal of psychological strategies<br>• Remote monitoring with feedback & action plans<br>• Adherence support & lifestyle interventions<br>•	Body functions:<br>• Improved pain, sustained for at least 6 months<br><br>Activities & Participation:<br>• Improved function, sustained for at least 6 months<br><br>Environment factors:<br>• Co-interventions & medication use was similar in both groups over the study period	
Internet-based with video conferencing	Hinman et al (2017)[35]<br><br>Qualitative	Chronic knee pain<br><br>N = 12<br><br>Age = 39	See Bennell el al (2017)[140]	Bennell el al (2017)[140]	Bennell el al (2017)[140]	• Overall positive experience using Skype as service delivery model for exercise management<br>• Feasible & acceptable & has potential to increase access to supervised exercise management	
**Key - Outcomes**							

Not applicable

Outcome improved post intervention

Outcome worsened post intervention

Outcome unchanged post intervention

Outcome had mixed results (some improved and/or some did not change and/or some worsened)

Abbreviations
ACT, Acceptance and Commitment Therapy; AIMS2, Arthritis Measurement Impact Scales-2; C, control; CB, cognitive behavioral; E, experimental; FM, fibromyalgia; HEP, home exercise programme; LBP, low back pain; IVR, interactive voice response; M, male; MDT, multidisciplinary team; MMR, multimodal pain rehabilitation; N/A, not applicable; OA, osteoarthritis; Obs, observational; OT, occupational therapist; PA, physical activity; PCP, Profile of Chronic Pain; PCST, pain coping skills training; PT, physiotherapy; QOL, quality of life; RCT, randomized controlled trial; TENS, transcutaneous electric nerve stimulation; TUAG, timed up and go; VAS, visual analogue scale; VR, virtual reality; Web-BCPA, Web Behaviour Change Program for Activity; WHO, world health organisation

References

1. James SL, Abate D, Abate KH, et al. Global, regional, and national incidence, prevalence, and years lived with disability for 354 diseases and injuries for 195 countries and territories, 1990–2017: a systematic analysis for the Global Burden of Disease Study 2017. *Lancet (London, England).* 2018;392(10159):1789-1858.

2. Briggs AM, Woolf AD, Dreinhöfer K, et al. Reducing the global burden of musculoskeletal conditions. *Bull World Health Organ.* 2018;96(5):366.

3. Blyth FM, Briggs AM, Schneider CH, Hoy DG, March LM. The global burden of musculoskeletal pain—where to from here? *Am J Public Health.* 2019;109(1):35-40.

4. Showell C, Nohr C. How should we define eHealth, and does the definition matter? *Stud Health Technol Inform.* 2012;180:881-884.

5. Gee PM, Greenwood DA, Paterniti DA, Ward D, Miller LMS. The eHealth enhanced chronic care model: a theory derivation approach. *Journal of medical Internet research.* 2015;17(4):e4067.

6. Cottrell MA, Russell TG. Telehealth for musculoskeletal physiotherapy. *Musculoskelet Sci Pract.* 2020:102193.

7. Heapy AA, Higgins DM, Cervone D, Wandner L, Fenton BT, Kerns RD. A Systematic Review of Technology-assisted Self-Management Interventions for Chronic Pain: Looking Across Treatment Modalities. *The Clinical journal of pain.* Jun 2015;31(6):470-492.

8. Wind TR, Rijkeboer M, Andersson G, Riper H. The COVID-19 pandemic: The 'black swan'for mental health care and a turning point for e-health. *Internet Interv.* 2020;20.

9. Tack C, Grodon J, Shorthouse F, Spahr N. "Physio anywhere": digitally-enhanced outpatient care as a legacy of coronavirus 2020. *Physiotherapy.* 2021.

10. Telehealth is here to stay. *Nat Med.* 2021/07/01 2021;27(7):1121-1121.

11. Devan H, Farmery D, Peebles L, Grainger R. Evaluation of self-management support functions in apps for people with persistent pain: systematic review. *JMIR mHealth and uHealth.* 2019;7(2):e13080.

12. Eccleston C, Fisher E, Brown R, et al. Psychological therapies (Internet-delivered) for the management of chronic pain in adults. *Cochrane Database Syst Rev.* 2014(2).

13. McGowan PT. Self-management education and support in chronic disease management. *Prim Care.* 2012;39(2):307-325.

14. Naylor C, Imison C, Addicott R, et al. Transforming our health care system: ten priorities for commissioners. *London: The Kings Fund.* 2015.

15. de Silva D. *Helping people help themselves: A review of the evidence considering whether it is worthwhile to support self-management*: The Health Foundation; 2011.

16. Mann EG, LeFort S, VanDenKerkhof EG. Self-management interventions for chronic pain. *Pain Manag.* 2013;3(3):211-222.

17. Janjua S, Banchoff E, Threapleton CJ, Prigmore S, Fletcher J, Disler RT. Digital interventions for the management of chronic obstructive pulmonary disease. *Cochrane Database Syst Rev.* 2021(4).

18. Fairbrother P, Pinnock H, Hanley J, et al. Exploring telemonitoring and self-management by patients with chronic obstructive pulmonary disease: a qualitative study embedded in a randomized controlled trial. *Patient Educ Couns.* 2013;93(3):403-410.

19. Taylor SJC, Pinnock H, Epiphaniou E, et al. *A rapid synthesis of the evidence on interventions supporting self-management for people with long-term conditions: PRISMS - Practical systematic Review of Self-Management Support for long-term conditions*. Southampton (UK); NIHR Journals Library2014.

20. Pearce G, Parke HL, Pinnock H, et al. The PRISMS taxonomy of self-management support: derivation of a novel taxonomy and initial testing of its utility. *J Health Serv Res Policy.* Apr 2016;21(2):73-82.

21. Sheridan N, Kenealy T, Kuluski K, McKillop A, Parsons J, Wong-Cornall C. Are patient and carer experiences mirrored in the Practice Reviews of Self-Management Support (PRISMS) provider taxonomy? *Int J Integr Care.* 2017;17(2).

22. Hanlon P, Daines L, Campbell C, McKinstry B, Weller D, Pinnock H. Telehealth interventions to support self-management of long-term conditions: a systematic metareview of diabetes, heart failure, asthma, chronic obstructive pulmonary disease, and cancer. *J Med Internet Res.* 2017;19(5):e172.

23. Pinnock H, Parke HL, Panagioti M, et al. Systematic meta-review of supported self-management for asthma: a healthcare perspective. *BMC Med.* Mar 17 2017;15(1):64.

24. Peters M, Godfrey C, McInerney P, Munn ZT, AC, Khalil H. Chapter 11: Scoping Reviews (2020 version). In: Aromataris E, Munn Z (Editors). JBI Manual for Evidence Synthesis, JBI, 2020. *JBI Manual for Evidence Synthesis [Internet]* 2020; JBI Manual for Evidence Synthesis [Internet]:JBI Manual for Evidence Synthesis [Internet]. Available at: Available from https://reviewersmanual.joannabriggs.org/. Accessed 07/01/21.

25. Page MJ, Moher D, Bossuyt PM, et al. PRISMA 2020 explanation and elaboration: updated guidance and exemplars for reporting systematic reviews. *BMJ.* 2021;372.

26. Kelly M, Fullen B, Martin D, McMahon S, McVeigh JG. eHealth interventions to support self-management in people with musculoskeletal disorders: a scoping review protocol. *JBI Evid Synth.* 2021;19(3):709-720.

27. CADTH. Grey Matters: A practical tool for searching health-related grey literature. Ottawa; 2019.

28. Hoffmann TC, Glasziou PP, Boutron I, et al. Better reporting of interventions: template for intervention description and replication (TIDieR) checklist and guide. *BMJ.* 2014;348:g1687.

29. WHO. *International classification of functioning, disability and health: ICF*: Geneva: World Health Organization; 2001.

30. Rini C, Porter LS, Somers TJ, et al. Automated, internet-based pain coping skills training to manage osteoarthritis pain: A randomized controlled trial. *Pain.* 2015;156(5):837.

31. Goode AP, Taylor SS, Hastings SN, Stanwyck C, Coffman CJ, Allen KD. Effects of a Home-Based Telephone-Supported Physical Activity Program for Older Adult Veterans With Chronic Low Back Pain. *Phys Ther.* 2018;98(5):369-380.

32. Wong YK, Hui E, Woo J. A community-based exercise programme for older persons with knee pain using telemedicine. *J Telemed Telecare.* 2005;11(6):310-315.

33. Guilkey RE, Draucker CB, Wu J, Yu Z, Kroenke K. Acceptability of a telecare intervention for persistent musculoskeletal pain. *J Telemed Telecare.* 2016;24(1):44-50.

34. Higgins DM, Buta E, Williams DA, et al. Internet-Based Pain Self-Management for Veterans: Feasibility and Preliminary Efficacy of the Pain EASE Program. *Pain Pract.* 2020.

35. Hinman R, Nelligan R, Bennell K, Delany C. "Sounds a bit crazy, but it was almost more personal:" a qualitative study of patient and clinician experiences of physical therapist–prescribed exercise for knee osteoarthritis via Skype. *Arthritis Care Res (Hoboken).* 2017;69(12):1834-1844.

36. Salisbury C, Foster NE, Hopper C, et al. A pragmatic randomised controlled trial of the effectiveness and cost-effectiveness of 'PhysioDirect' telephone assessment and advice services for physiotherapy. *Health Technol Assess.* 2013;17(2):1-157.

37. Hasenöhrl T, Windschnurer T, Dorotka R, Ambrozy C, Crevenna R. Prescription of individual therapeutic exercises via smartphone app for patients suffering from non-specific back pain. *Wien Klin Wochenschr.* 2020:1-9.

38. Hinman RS, Delany CM, Campbell PK, Gale J, Bennell KL. Physical therapists, telephone coaches, and patients with knee osteoarthritis: qualitative study about working together to promote exercise adherence. *Phys Ther.* 2016;96(4):479-493.

39. Kristjánsdóttir ÓB, Fors EA, Eide E, et al. Written online situational feedback via mobile phone to support self-management of chronic widespread pain: a usability study of a Web-based intervention. *BMC Musculoskelet Disord.* 2011;12:51-51.

40. Petrozzi MJ, Leaver A, Ferreira PH, Rubinstein SM, Jones MK, Mackey MG. Addition of MoodGYM to physical treatments for chronic low back pain: a randomized controlled trial. *Chiropr Man Therap.* 2019;27(1).

41. Chhabra H, Sharma S, Verma S. Smartphone app in self-management of chronic low back pain: a randomized controlled trial. *Eur Spine J.* 2018;27(11):2862-2874.

42. Malliaras P, Merolli M, Williams C, Caneiro J, Haines T, Barton C. 'It's not hands-on therapy, so it's very limited': Telehealth use and views among allied health clinicians during the coronavirus pandemic. *Musculoskelet Sci Pract.* 2021;52:102340.

43. Rausch A-K, Baur H, Reicherzer L, et al. Physiotherapists' use and perceptions of digital remote physiotherapy during COVID-19 lockdown in Switzerland: an online cross-sectional survey. *Archives of physiotherapy.* 2021;11(1):1-10.

44. Werneke MW, Deutscher D, Grigsby D, Tucker CA, Mioduski JE, Hayes D. Telerehabilitation during the Covid-19 pandemic in outpatient rehabilitation settings: a descriptive study. *Physical therapy.* 2021;101(7):pzab110.

45. Slattery BW, Haugh S, O'Connor L, et al. An Evaluation of the Effectiveness of the Modalities Used to Deliver Electronic Health Interventions for Chronic Pain: Systematic Review With Network Meta-Analysis. *J Med Internet Res.* 2019;21(7):e11086.

46. Du S, Liu W, Cai S, Hu Y, Dong J. The efficacy of e-health in the self-management of chronic low back pain: A meta analysis. *Int J Nurs Stud.* 2020;106:103507.

47. Dario AB, Cabral AM, Almeida L, et al. Effectiveness of telehealth-based interventions in the management of non-specific low back pain: a systematic review with meta-analysis. *Spine J.* 2017;17(9):1342-1351.

48. Kruse C, Karem P, Shifflett K, Vegi L, Ravi K, Brooks M. Evaluating barriers to adopting telemedicine worldwide: A systematic review. *J Telemed Telecare.* 2018;24(1):4-12.

49. Tanne JH, Hayasaki E, Zastrow M, Pulla P, Smith P, Rada AG. Covid-19: how doctors and healthcare systems are tackling coronavirus worldwide. *Bmj.* 2020;368.

50. K M. Telehealth in the time of coronavirus [Internet]. Pulse+IT. 2020 [accessed 21 July 2021]. Available from: https://www.pulseitmagazine.com.au/blog/5399-telehealth-in-the-time-of-coronavirus. 2020.

51. Herbert MS, Afari N, Liu L, et al. Telehealth versus in-person acceptance and commitment therapy for chronic pain: a randomized noninferiority trial. *J Pain.* 2017;18(2):200-211.

52. Kress H-G, Aldington D, Alon E, et al. A holistic approach to chronic pain management that involves all stakeholders: change is needed. *Curr Med Res Opin.* 2015;31(9):1743-1754.

53. Cranen K, Groothuis-Oudshoorn CG, Vollenbroek-Hutten MM, IJzerman MJ. Toward patient-centered telerehabilitation design: understanding chronic pain patients' preferences for web-based exercise telerehabilitation using a discrete choice experiment. *J Med Internet Res.* 2017;19(1):e26.

54. Michie S, Yardley L, West R, Patrick K, Greaves F. Developing and evaluating digital interventions to promote behavior change in health and health care: recommendations resulting from an international workshop. *Journal of medical Internet research.* 2017;19(6):e7126.

55. Lo WLA, Lei D, Li L, Huang DF, Tong K-F. The Perceived Benefits of an Artificial Intelligence–Embedded Mobile App Implementing Evidence-Based Guidelines for the Self-Management of Chronic Neck and Back Pain: Observational Study. *JMIR mHealth and uHealth.* 2018;6(11):e198.

56. Ljótsson B, Atterlöf E, Lagerlöf M, et al. Internet-delivered acceptance and values-based exposure treatment for fibromyalgia: a pilot study. *Cogn Behav Ther.* 2014;43(2):93-104.

57. Williams DA, Kuper D, Segar M, Mohan N, Sheth M, Clauw DJ. Internet-enhanced management of fibromyalgia: a randomized controlled trial. *Pain.* 2010;151(3):694-702.

58. Friesen LN, Hadjistavropoulos HD, Schneider LH, Alberts NM, Titov N, Dear BF. Examination of an internet-delivered cognitive behavioural pain management course for adults with fibromyalgia: a randomized controlled trial. *Pain.* 2017;158(4):593-604.

59. Naylor MR, Helzer JE, Naud S, Keefe FJ. Automated telephone as an adjunct for the treatment of chronic pain: a pilot study. *J Pain.* Dec 2002;3(6):429-438.

60. Hauser-Ulrich S, Künzli H, Meier-Peterhans D, Kowatsch T. A smartphone-based health care chatbot to promote self-management of chronic pain (SELMA): pilot randomized controlled trial. *JMIR Mhealth Uhealth.* 2020;8(4):e15806.

61. Naylor MR, Keefe FJ, Brigidi B, Naud S, Helzer JE. Therapeutic Interactive Voice Response for chronic pain reduction and relapse prevention. *Pain.* 2008;134(3):335-345.

62. Naylor MR, Naud S, Keefe FJ, Helzer JE. Therapeutic Interactive Voice Response (TIVR) to reduce analgesic medication use for chronic pain management. *J Pain.* 2010;11(12):1410-1419.

63. Ruehlman LS, Karoly P, Enders C. A randomized controlled evaluation of an online chronic pain self management program. *Pain.* Feb 2012;153(2):319-330.

64. Buhrman M, Nilsson-Ihrfeldt E, Jannert M, Ström L, Andersson G. Guided internet-based cognitive behavioural treatment for chronic back pain reduces pain catastrophizing: a randomized controlled trial. *J Rehabil Med.* 2011;43(6):500-505.

65. Buhrman M, Skoglund A, Husell J, et al. Guided internet-delivered acceptance and commitment therapy for chronic pain patients: a randomized controlled trial. *Behav Res Ther.* 2013;51(6):307-315.

66. Dear BF, Courtney, C., Khor, K. E., McDonald, S., Ricciardi, T., Gandy, M., Fogliati, V. J., & Titov, N. The Pain Course: Exploring the Feasibility of an Internet-delivered Pain Management Program When Offered by a Tertiary Pain Management Service. *Clin J Pain.* 2018;34:505–514.

67. Dear BF, Gandy M, Karin E, et al. The Pain Course: 12-and 24-Month Outcomes From a Randomized Controlled Trial of an Internet-Delivered Pain Management Program Provided With Different Levels of Clinician Support. *J Pain.* 2018;19(12):1491-1503.

68. Dear BF, Gandy M, Karin E, et al. The Pain Course: a randomised controlled trial examining an internet-delivered pain management program when provided with different levels of clinician support. *Pain.* 2015;156(10):1920.

69. Dear BF, Titov N, Perry KN, et al. The Pain Course: a randomised controlled trial of a clinician-guided Internet-delivered cognitive behaviour therapy program for managing chronic pain and emotional well-being. *Pain.* 2013;154(6):942-950.

70. Kristjánsdóttir ÓB, Fors EA, Eide E, et al. A smartphone-based intervention with diaries and therapist-feedback to reduce catastrophizing and increase functioning in women with chronic widespread pain: randomized controlled trial. *J Med Internet Res.* 2013;15(1):e5.

71. Kristjánsdóttir ÓB, Fors EA, Eide E, et al. A smartphone-based intervention with diaries and therapist feedback to reduce catastrophizing and increase functioning in women with chronic widespread pain. part 2: 11-month follow-up results of a randomized trial. *J Med Internet Res.* 2013;15(3):e72.

72. Kroenke K, Baye F, Lourens SG, et al. Automated Self-management (ASM) vs. ASM-Enhanced Collaborative Care for Chronic Pain and Mood Symptoms: the CAMMPS Randomized Clinical Trial. *J Gen Intern Med.* 2019.

73. Kroenke K, Krebs EE, Wu J, Yu Z, Chumbler NR, Bair MJ. Telecare collaborative management of chronic pain in primary care: a randomized clinical trial. *JAMA.* 2014;312(3):240-248.

74. Buhrman M, Syk M, Burvall O, Hartig T, Gordh T, Andersson G. Individualized Guided Internet-delivered Cognitive-Behavior Therapy for Chronic Pain Patients With Comorbid Depression and Anxiety A Randomized Controlled Trial. *Clin J Pain.* Jun 2015;31(6):504-516.

75. Trompetter HR, Bohlmeijer ET, Veehof MM, Schreurs KM. Internet-based guided self-help intervention for chronic pain based on Acceptance and Commitment Therapy: a randomized controlled trial. *J Behav Med.* 2015;38(1):66-80.

76. Wilson M, Roll JM, Corbett C, Barbosa-Leiker C. Empowering patients with persistent pain using an internet-based self-management program. Pain Manag Nurs. 2015;16(4):503-514.

77. Nordin CA, Michaelson P, Gard G, Eriksson MK. Effects of the web behavior change program for activity and multimodal pain rehabilitation: randomized controlled trial. J Med Internet Res. 2016;18(10):e265.

78. Calner T, Nordin C, Eriksson MK, Nyberg L, Gard G, Michaelson P. Effects of a self- guided, web-based activity programme for patients with persistent musculoskeletal pain in primary healthcare: A randomized controlled trial. Eur J Pain. 2017;21(6):1110-1120.

79. Lin J, Paganini S, Sander L, et al. An internet-based intervention for chronic pain: a three-arm randomized controlled study of the effectiveness of guided and unguided acceptance and commitment therapy. Dtsch Arztebl Int. 2017;114(41):681.

80. Nes AAG, van Dulmen S, Wicksell R, Fors EA, Eide H. Analyzing Change Processes Resulting from a Smartphone Maintenance Intervention Based on Acceptance and Commitment Therapy for Women with Chronic Widespread Pain. Int J Behav Med. Apr 2017;24(2):215-229.

81. Peters ML, Smeets E, Feijge M, et al. Happy despite pain: a randomized controlled trial of an 8-week internet-delivered positive psychology intervention for enhancing well-being in patients with chronic pain. Clin J Pain. 2017;33(11):962.
82. Johnson SS, Levesque DA, Broderick LE, Bailey DG, Kerns RD. Pain selfmanagement for veterans: development and pilot test of a stage-based mobileoptimized intervention. JMIR Med Inform. 2017;5(4):e40.
83. Kamaleri Y, Natvig B, Ihlebaek CM, Bruusgaard D. Localized or widespread musculoskeletal pain: does it matter? Pain. 2008;138(1):41-46.
84. Nicholl BI, Sandal LF, Stochkendahl MJ, et al. Digital support interventions for the self-management of low back pain: a systematic review. J Med Internet Res. 2017;19(5):e179.
85. Oliveira VC, Ferreira PH, Maher CG, Pinto RZ, Refshauge KM, Ferreira ML. Effectiveness of self-management of low back pain: Systematic review with meta- analysis. Arthritis Care Res (Hoboken). 2012;64(11):1739-1748.
86. NICE. Low back pain and sciatica in over 16s: assessment and management. Available at https://www.nice.org.uk/guidance/ng59. 2016.
87. Henschke N, Maher CG, Refshauge KM, et al. Prognosis in patients with recent onset low back pain in Australian primary care: inception cohort study. BMJ. Jul 7 2008;337:a171.
88. Hancock MJ, Maher CG, Latimer J, Herbert RD, McAuley JH. Can rate of recovery be predicted in patients with acute low back pain? Development of a clinical prediction rule. Eur J Pain. 2009;13(1):51-55.
89. Chou R, Shekelle P. Will this patient develop persistent disabling low back pain? Jama. Apr 7 2010;303(13):1295-1302.
90. Maher C, Underwood M, Buchbinder R. Non-specific low back pain. The Lancet. 2017;389(10070):736-747.
91. Panagioti M, Richardson G, Small N, et al. Self-management support interventions to reduce health care utilisation without compromising outcomes: a systematic review and meta-analysis. BMC Health Serv Res. 2014;14(1):356.
92. Garg S, Garg D, Turin TC, Chowdhury MFU. Web-based interventions for chronic back pain: A systematic review. J Med Internet Res. 2016;18(7):52-64.
93. Munn Z, Peters MD, Stern C, Tufanaru C, McArthur A, Aromataris E. Systematic review or scoping review? Guidance for authors when choosing between a systematic or scoping review approach. BMC Med Res Methodol. 2018;18(1):1-7.
94. Eysenbach G, Group C-E. CONSORT-EHEALTH: improving and standardizing evaluation reports of Web-based and mobile health interventions. J Med Internet Res. 2011;13(4):e126.
95. Chiauzzi E, Pujol LA, Wood M, et al. painACTION-back pain: a self-management website for people with chronic back pain. Pain Med. 2010;11(7):1044-1058.
96. Jelin E, Granum V, Eide H. Experiences of a web-based nursing intervention-- interviews with women with chronic musculoskeletal pain. Pain Management Nursing: Official Journal Of The American Society Of Pain Management Nurses. 2012;13(1):2-10.
97. Carpenter KM, Stoner SA, Mundt JM, Stoelb B. An online self-help CBT intervention for chronic lower back pain. Clin J Pain. 2012;28(1):14-22.
98. Wilson M, Shaw MR. Participant perspectives on benefits and challenges of engaging in an online pain self-management program. Int J Healthc Inf Syst Inform. 2017;12(4):52-67.

99. Bossen D, Veenhof C, Van Beek KE, Spreeuwenberg PM, Dekker J, De Bakker DH. Effectiveness of a web-based physical activity intervention in patients with knee and/or hip osteoarthritis: randomized controlled trial. J Med Internet Res. 2013;15(11):e257.
100. Bossen D, Buskermolen M, Veenhof C, de Bakker D, Dekker J. Adherence to a webbased physical activity intervention for patients with knee and/or hip osteoarthritis: A mixed method study. J Med Internet Res. 2013;15(10):55-66.
101. Buhrman M, Fredriksson A, Edstrom G, et al. Guided Internet-delivered cognitive behavioural therapy for chronic pain patients who have residual symptoms after rehabilitation treatment: Randomized controlled trial. Eur J Pain. May 2013;17(5):753-765.
102. Krein SL, Kadri R, Hughes M, et al. Pedometer-based internet-mediated intervention for adults with chronic low back pain: randomized controlled trial. J Med Internet Res. 2013;15(8):e181.
103. Riva S, Camerini A-L, Allam A, Schulz PJ. Interactive sections of an Internet-based intervention increase empowerment of chronic back pain patients: randomized controlled trial. J Med Internet Res. 2014;16(8):e180-e180.
104. Irvine AB, Russell H, Manocchia M, et al. Mobile-Web app to self-manage low back pain: randomized controlled trial. J Med Internet Res. 2015;17(1):e1.
105. Nordin C, Michaelson P, Eriksson MK, Gard G. It's about me: Patients' experiences of patient participation in the web behavior change program for activity in combination with multimodal pain rehabilitation. J Med Internet Res. 2017;19(1).
106. Pearson J, Walsh N, Carter D, Koskela S, Hurley M. Developing a Web-Based Version of An Exercise-Based Rehabilitation Program for People With Chronic Knee and Hip Pain: A Mixed Methods Study. JMIR Res Protoc. 2016;5(2):e67-e67.
107. Bendelin N, Gerdle B, Andersson G. Internet-delivered aftercare following multimodal rehabilitation program for chronic pain: a qualitative feasibility study. J Pain Res. 2018;11:1715-1728.
108. Bennell KL, Nelligan RK, Rini C, et al. Effects of internet-based pain coping skills training before home exercise for individuals with hip osteoarthritis (HOPE trial): a randomised controlled trial. Pain. 2018;159(9):1833-1842.
109. Schlicker S, Baumeister H, Buntrock C, et al. A Web-and Mobile-Based Intervention for Comorbid, Recurrent Depression in Patients With Chronic Back Pain on Sick Leave (Get. Back): Pilot Randomized Controlled Trial on Feasibility, User Satisfaction, and Effectiveness. JMIR mental health. 2020;7(4):e16398.
110. Thomas KS, Muir KR, Doherty M, et al. Home based exercise programme for knee pain and knee osteoarthritis: randomised controlled trial. BMJ. Oct 2002;325(7367):752-755.
111. Thomas KS, Miller P, Doherty M, Muir KR, Jones AC, O'Reilly SC. Cost effectiveness of a two-year home exercise program for the treatment of knee pain. Arthritis Rheum. Jun 2005;53(3):388-394.
112. Damush TM, Weinberger M, Perkins SM, et al. Randomized trial of a selfmanagement program for primary care patients with acute low back pain: short-term effects. Arthritis Rheum. 2003;49(2):179-186.
113. Damush TM, Weinberger M, Perkins SM, et al. The long-term effects of a selfmanagement program for inner-city primary care patients with acute low back pain. Arch Intern Med. 2003;163(21):2632-2638.

114. Allen KD, Oddone EZ, Coffman CJ, et al. Telephone-based self-management of osteoarthritis: a randomized trial. Ann Intern Med. 2010;153(9):570-579.
115. Iles R, Taylor NF, Davidson M, O'Halloran P. Telephone coaching can increase activity levels for people with non-chronic low back pain: a randomised trial. J Physiother. 2011;57(4):231-238.
116. Odole AC, Ojo OD. A telephone-based physiotherapy intervention for patients with osteoarthritis of the knee. Int J Telerehabil. 2013;5(2):11.
117. Odole AC, Ojo OD. Is telephysiotherapy an option for improved quality of life in patients with osteoarthritis of the knee? Int J Telemed Appl. 2014;2014.
118. Bennell KL, Campbell PK, Egerton T, et al. Telephone coaching to enhance a home- based physical activity program for knee osteoarthritis: a randomized clinical trial. Arthritis Care Res (Hoboken). 2017;69(1):84-94.
119. Schaller A, Petrowski K, Pfoertner T-K, Froboese I. Effectiveness of a theory-based multicomponent intervention (Movement Coaching) on the promotion of total and domain-specific physical activity: a randomised controlled trial in low back pain patients. BMC Musculoskelet Disord. 2017;18(1):1-14.
120. Rutledge T, Atkinson JH, Chircop-Rollick T, et al. Randomized Controlled Trial of Telephone-delivered Cognitive Behavioral Therapy Versus Supportive Care for Chronic Back Pain. Clin J Pain. 2018;34(4):322-327.
121. Swoboda M. Telephonic health coaching for chronic low back pain. Coaching: An International Journal of Theory, Research and Practice. 2019;12(1):29-38. 122. Wang YY, Lombard C, Hussain SM, et al. Effect of a low-intensity, self-management lifestyle intervention on knee pain in community-based young to middle-aged rural women: a cluster randomised controlled trial. Arthritis Res Ther. Apr 2018;20.
123. Williams A, Wiggers J, O'Brien KM, et al. Effectiveness of a healthy lifestyle intervention for chronic low back pain: a randomised controlled trial. Pain. 2018;159(6):1137-1146.
124. Williams A, van Dongen JM, Kamper SJ, et al. Economic evaluation of a healthy lifestyle intervention for chronic low back pain: A randomized controlled trial. Eur J Pain. 2019. 2019;23(3):621-634.
125. Gialanella B, Comini L, Olivares A, Gelmini E, Ubertini E, Grioni G. Pain, disability and adherence to home exercises in patients with chronic neck pain: long term effects of phone surveillance. A randomized controlled study. Eur J Phys Rehabil Med. 2019.
126. Hinman RS, Campbell PK, Lawford BJ, et al. Does telephone-delivered exercise advice and support by physiotherapists improve pain and/or function in people with knee osteoarthritis? Telecare randomised controlled trial. Br J Sports Med. 2019.
127. Buhrman M, Fältenhag S, Ström L, Andersson G. Controlled trial of Internet-based treatment with telephone support for chronic back pain. Pain. 2004;111(3):368-377.
128. Bendelin N, Björkdahl P, Risell M, et al. Patients’ experiences of internet-based Acceptance and commitment therapy for chronic pain: a qualitative study. BMC Musculoskelet Disord. 2020;21:1-12.
129. Geraghty AWA, Stanford R, Stuart B, et al. Using an internet intervention to support self-management of low back pain in primary care: findings from a randomised controlled feasibility trial (SupportBack). BMJ open. 2018;8(3):e016768.

130. Geraghty A, Roberts L, Stanford R, et al. Exploring Patients' Experiences of InternetBased Self-Management Support for Low Back Pain in Primary Care. Pain Med. 2019.
131. Amorim AB, Pappas E, Simic M, et al. Integrating Mobile-health, health coaching, and physical activity to reduce the burden of chronic low back pain trial (IMPACT): a pilot randomised controlled trial. BMC Musculoskelet Disord. 2019;20(1):71.
132. Lee J, Lee M, Lim T, et al. Effectiveness of an application-based neck exercise as a pain management tool for office workers with chronic neck pain and functional disability: a pilot randomized trial. European Journal of Integrative Medicine. 2017;12:87-92.
133. Mecklenburg G, Smittenaar P, Erhart-Hledik JC, Perez DA, Hunter S. Effects of a 12- Week Digital Care Program for Chronic Knee Pain on Pain, Mobility, and Surgery Risk: randomized Controlled Trial. J Med Internet Res. 2018;20(4):e156.
134. Shebib R, Bailey JF, Smittenaar P, Perez DA, Mecklenburg G, Hunter S. Randomized controlled trial of a 12-week digital care program in improving low back pain. NPJ Digit Med. 2019;2(1).
135. Toelle TR, Utpadel-Fischler DA, Haas KK, Priebe JA. App-based multidisciplinary back pain treatment versus combined physiotherapy plus online education: a randomized controlled trial. NPJ Digit Med. 2019;2(1).
136. Huber S, Priebe JA, Baumann K-M, Plidschun A, Schiessl C, Tölle TR. Treatment of low back pain with a digital multidisciplinary pain treatment app: short-term results. JMIR Rehabil Assist Technol. 2017;4(2):e11.
137. Yang JY, Wei Q, Ge YL, Meng LJ, Zhao MD. Smartphone-Based Remote SelfManagement of Chronic Low Back Pain: A Preliminary Study. J Healthc Eng. 2019.
138. Heapy AA, Higgins DM, Goulet JL, et al. Interactive voice response–based selfmanagement for chronic back pain: the COPES noninferiority randomized trial. JAMA Intern Med. 2017;177(6):765-773.
139. Darnall BD, Krishnamurthy P, Tsuei J, Minor JD. Self-Administered Skills-Based Virtual Reality Intervention for Chronic Pain: Randomized Controlled Pilot Study. JMIR Form Res. 2020;4(7):e17293.
140. Bennell KL, Nelligan R, Dobson F, et al. Effectiveness of an internet-delivered exercise and pain-coping skills training intervention for persons with chronic knee pain: a randomized trial. Ann Intern Med. 2017;166(7):453-462.
